# Self-recruited neutrophils trigger over-activated innate immune response and phenotypic change of cardiomyocytes in fulminant viral myocarditis

**DOI:** 10.1038/s41421-023-00593-5

**Published:** 2023-10-10

**Authors:** Huihui Li, Mingzhi Zhang, Quanyi Zhao, Wanqing Zhao, Yan Zhuang, Jin Wang, Weijian Hang, Zheng Wen, Li Wang, Chen Chen, Dao Wen Wang

**Affiliations:** 1 Division of Cardiology, Department of Internal Medicine and Hubei Key Laboratory of Genetics and Molecular Mechanisms of Cardiological Disorders, Tongji Hospital, Tongji Medical College, Huazhong University of Science and Technology, Wuhan, Hubei China; 2 State Key Laboratory of Cardiovascular Disease, Fuwai Hospital, National Center for Cardiovascular Diseases, Chinese Academy of Medical Sciences and Peking Union Medical College, Beijing, China

**Keywords:** Transcriptomics, Innate immunity

## Abstract

Fulminant myocarditis (FM) is a life-threatening inflammatory disease. However, the mechanisms underlying its acute onset are unknown. By dynamic cardiac function measurement, we discovered that the initiation of sudden hemodynamic collapse was on day 4 in the mouse model of FM. Single-cell RNA-sequencing study revealed that healthy cardiomyocytes (CMs) lost their contractile and metabolic function and differentiated into pro-angiogenic and pro-inflammatory CMs. Meanwhile, neutrophils, the most expanded immune cells, exhibited a unique developmental trajectory only after migrating to the heart, where they continuously attracted peripheral neutrophils via Cxcl2/Cxcl3, resulting in the acute accumulation of neutrophils in the heart. Well-differentiated cardiac-infiltrating neutrophils, rather than viruses, induced phenotypic changes in CMs. Moreover, neutrophils could amplify cytokine storm by recruiting and activating pro-inflammatory monocytes. Blockade of the self-recruiting loop of neutrophils by targeting the Cxcl2/Cxcl3-Cxcr2 axis substantially alleviated FM in mice. Collectively, we provide a comprehensive single-cell atlas of immune cells and CMs in FM, elucidate the disease pathogenesis, and suggest potential therapeutic strategies.

## Introduction

Myocarditis is an inflammatory disease primarily caused by viral infections or other pathogens, such as bacteria, protozoans, and fungi, as well as numerous toxic substances, drugs, and systemic immune-mediated diseases^1–3^. Fulminant myocarditis (FM) is a distinct form of myocarditis characterized by a sudden onset and rapid deterioration of cardiac function, which requires inotropic and mechanical circulatory support^1,4,5^. Both the direct cytopathic effects of the pathogen and immune response-mediated myocardial injury are responsible for cardiac pathology in myocarditis^6–8^, and an over-activated immune response is speculated to play a central role in the development of FM^1^.

Despite advances in FM pathology, the fundamental and systemic immunological signatures associated with FM remain largely unknown^3^. Moreover, the contribution of various types of immune cells to the inflammatory responses during FM progression remains unclear. However, cell–cell interactions in the immune microenvironment of FM are difficult to study because of the complex network of cardiac and immune cell types and cellular heterogeneity. A limited understanding of this disease hinders the development of early diagnostic tools and effective treatment strategies. Previous research demonstrated a heterogeneous immunological environment in both experimental autoimmune myocarditis mouse model and patients with checkpoint inhibitor-induced myocarditis at single-cell resolution and suggested complex dysregulation of peripheral and cardiac immune responses^9,10^, highlighting the need to completely elucidate the pathogenesis of myocarditis with different aetiologies. Besides, in contrast to the broad application of single-cell sequencing in developmental studies, the single-cell-level analysis of adult cardiomyocytes (CMs) is hampered by their large size. Consequently, neither functional changes in CMs during disease progression nor the immune cell types responsible for myocardial damage have been identified. In addition, current immunomodulatory therapies, such as glucocorticoids and intravenous immunoglobulin, are largely nonselective and have non-specific side effects^11^, emphasizing the need for a deeper understanding of the cellular subsets and mechanisms implicated in FM.

This study aimed to establish a single-cell landscape with pronounced resolution at different time points and gain a comprehensive insight into the immune response during the progression of FM by analyzing single-cell RNA-sequencing (scRNA-seq) data of CMs, immune cells that infiltrate cardiac tissues, and peripheral immune cells of mice with FM. We focused predominantly on neutrophils, which significantly increased when cardiac dysfunction was initiated. The detailed molecular signatures identified in our study contribute to a better understanding of the mechanisms underlying sudden cardiac function collapse in FM and provide novel treatment strategies for patients with FM.

## Results

### Cardiac dysfunction of mice with FM initiates on day 4

**Fig. 1:**
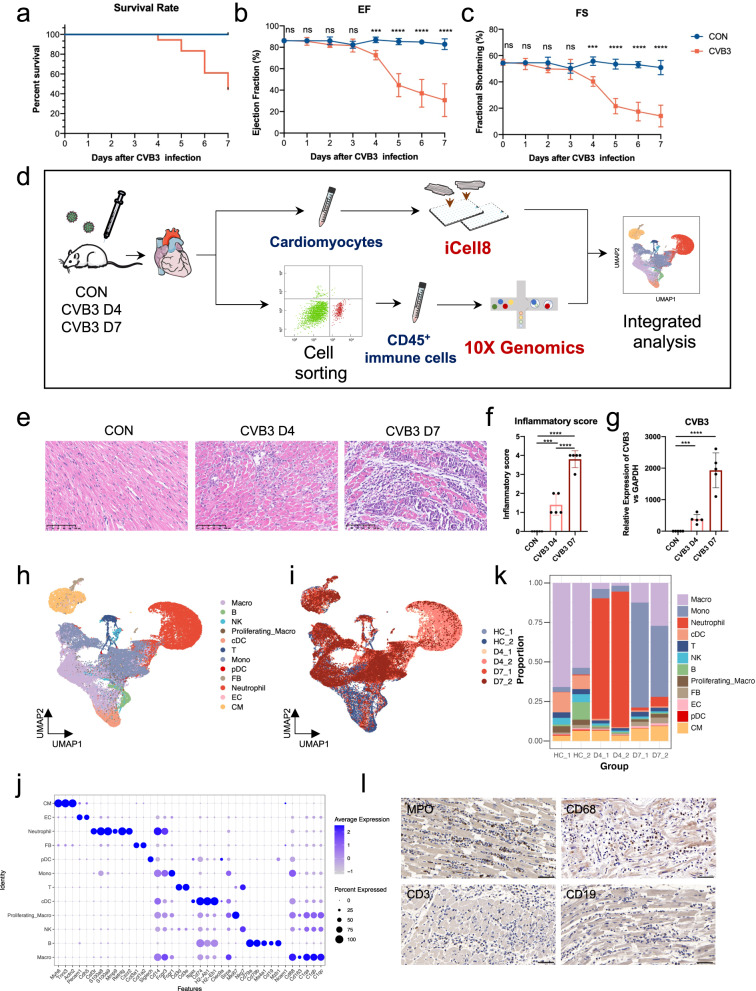
Cardiac cell subset diversity in FM mice at different time points. **a**–**c** Survival rate (**a**), ejection fraction (**b**), and fraction shortening (**c**) changes of FM mice during disease progression. **d** Schematic diagram of the workflow for sample processing and scRNA-seq. **e** Cardiac immune infiltration status among different groups. Scale bars: 100 μm. **f** Inflammatory score of FM mice among different groups. **g** quantitative PCR (qPCR) of cardiac CVB3 levels in different groups. **h**, **i** UMAP embedding of all the cardiomyocytes and immune cells colored by manually annotated cell types (**h**) and samples (**i**). **j** Bubble plot showing the expression levels of cell-typing genes in each cell type. The dot color represents the gene expression level, and the dot size represents the percentage of cells expressing the respective gene. **k** Cell component changes among different time points. **l** Immunohistochemical staining of cardiac autopsy samples from patients with FM. Scale bars: 50 μm.

To elucidate the pathogenesis underlying sudden cardiac collapse in FM, an A/J mouse model of FM was established using coxsackievirus B3 (CVB3) injection^12^. The mortality rate of the FM mice was approximately 60% on day 7 (Fig. 1a). Furthermore, all mice injected with CVB3 exhibited a significant decrease in body weight (Supplementary Fig. S1a), reflecting a poor general health status. We measured the dynamic changes in cardiac function during disease progression (Fig. 1b, c and Supplementary Fig. S1b–h). As expected, the cardiac function of mice with FM significantly deteriorated during the acute and severe inflammatory stage (from day 0 to day 7) (Fig. 1b, c and Supplementary Fig. S1b–d), similar to the rapid deterioration of cardiac function observed in patients with FM^13^. Intriguingly, we observed that the systolic dysfunction was initiated on day 4, followed by a sudden collapse of cardiac function (Fig. 1b, c and Supplementary Fig. S1b). Hence, day 4 may be an important time point for FM progression. In addition, cardiac output and stroke volume decreased on day 1 (Supplementary Fig. S1c, d), which may be attributed to the general muscle loss and diminished heart volume (Supplementary Fig. S1b, e–h).

### scRNA-seq and cell typing of cardiomyocytes and immune cells in mice with FM at different time points

Cardiac systolic function was exacerbated on day 4, whereas the first 7 days were characterized by an acutely severe inflammatory stage of CVB3-induced myocarditis^14^. Thus, CMs and immune cells were isolated on days 0, 4, and 7, and subjected to scRNA-seq (Fig. 1d). Similarly, mice injected with CVB3 exhibited a high death rate, decreased body and heart weights (Supplementary Fig. S2a–c) and a progressive decline in left ventricular function during disease progression (Supplementary Fig. S2d–g). Cardiac infiltration of inflammatory cells was sparse on day 4 and became severe on day 7 (Fig. 1e, f). The relative level of CVB3 in the heart increased in a time-dependent manner (Fig. 1g).

In total, 4068 CMs (Supplementary Fig. S3a) and 52,711 immune cells (Supplementary Fig. S3b) passed strict quality control and were used for further analysis. We manually annotated cell clusters based on their respective cellular identities (Supplementary Fig. S3c–f). After cell integration, an average of 9463 cells were obtained per sample (Supplementary Fig. S3g), with median gene and mRNA numbers of 1566 and 4116, respectively (Supplementary Fig. S3h, i). Twelve cell types were acquired (Fig. 1h, i and Supplementary Fig. S3j) and each cell type expressed representative marker genes (Fig. 1j and Supplementary Fig. S3k and Table S1). Calculation of the cell proportion for each cell type indicated a significant time-dependent change (Fig. 1k). Cardiac immune cell heterogeneity is observed under homeostatic conditions, and the macrophage population is the most abundant, accounting for approximately 70% of immune cells. Consistent with previous reports, the immune cells in normal hearts are predominantly tissue-resident macrophages^15,16^. Apparent alterations in cluster proportions were observed on day 7 after CVB3 injection when the proportion of monocytes increased significantly (Fig. 1k). This finding is consistent with that of previous study in which peripheral monocytes egressed from the bone marrow were recruited to the inflammatory heart site and exerted a pro-inflammatory effect during the acute inflammatory stage^17,18^.

However, the neutrophil population exhibited a remarkable expansion on day 4 after CVB3 injection, accounting for > 80% of the immune cells at this time point (Fig. 1k). This indicated that neutrophils play a significant role in the initiation of cardiac dysfunction. Both T and B lymphocytes are present in small proportions during disease progression. These observations were confirmed by immunohistochemical staining (Supplementary Fig. S2h). Although the inflammatory infiltration of the heart on day 4 was relatively mild, neutrophils were the predominant immune cells. Simmilar phenomenon was also observed in autopsy samples of patients with FM who experienced sudden death, wherein substantial infiltration of neutrophils was observed, followed by macrophages, whereas T and B cells were sparse (Fig. 1l).

These results highlight the importance of the innate immune response to FM, rather than the adaptive immune response. In addition, the population of immune cells infiltrating the heart differs at different time points, indicating the dynamic progression of the disease. Moreover, neutrophils may play a significant role in the early stages of cardiac dysfunction.

### CMs exhibit profound transcriptional change before the collapse of cardiac function

**Fig. 2:**
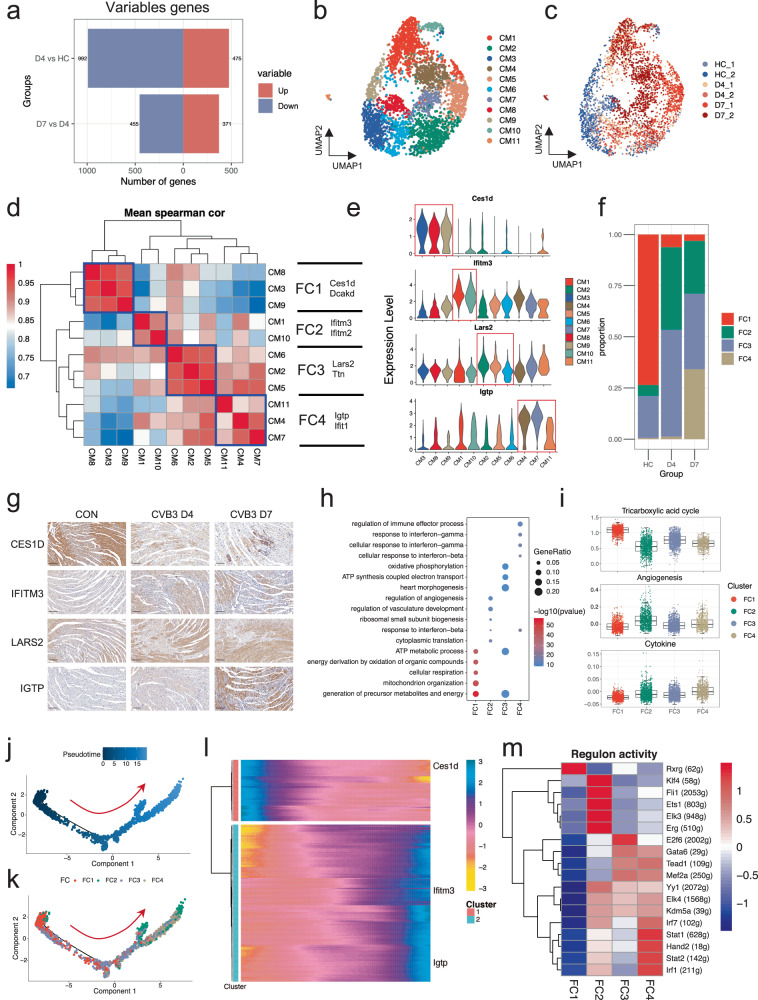
Characterization of cardiomyocytes at different stages of FM. **a** Numbers of up- and down-regulated genes of CMs at different time points. **b**, **c** UMAP embedding of all the CMs colored by clusters (**b**) and samples (**c**). **d** 11 CM subtypes are grouped into 4 FCs by Spearman correlation analysis. FC1 = CM3 + CM8 + CM9, FC2 = CM1 + CM10, FC3 = CM2 + CM5 + CM6, FC4 = CM4 + CM7 + CM11. **e** Expression of marker genes for each FC. **f** Cell proportion change of FCs at different time points. **g** Immunohistochemical staining of the marker genes of each FCs at different time points. Scale bars: 100 μm. **h** Gene Ontology (GO) analysis of specifically expressed genes in each FC. **i** Box plot of the cardiac tricarboxylic acid cycle, angiogenesis, and cytokine scores among different FCs. **j**, **k** Developmental trajectories of FC1, FC2, FC3, and FC4 colored by pseudotime (**j**) and FCs (**k**). **l** Expression of marker genes for each FC along the developmental trajectory. **m** Heatmap of regulon activity in each FC.

The CMs were characterized by the expression of myosin heavy chain 6 (Myh6), troponins (Tnni3), and cytoskeletal proteins (Actn2) (Fig. 1j)^19^. Because cardiac systolic dysfunction is a typical symptom of patients with FM, we first analyzed transcriptional changes in CMs. We identified differentially expressed genes (DEGs) by comparing CMs at different time points (Supplementary Fig. S4a, b). Notably, several interferon- and immune-associated genes showed time-dependent increases in gene expression during disease progression (Supplementary Fig. S4c), consistent with the characteristics associated with virus-induced diseases^20^. The transcriptional factors (TFs) that modulate CMs at different time points also changed (Supplementary Fig. S4d). Surprisingly, we discovered that the CMs underwent more drastic transcriptional changes between days 0 and 4 than between days 4 and 7 (Fig. 2a). Further enrichment analysis revealed that although metabolic disorders, particularly fatty acid metabolism, were more severe on day 7, several metabolic pathways had been dysregulated by day 4 (Supplementary Fig. S4e). Besides, the genes downregulated on day 4 were mainly associated with metabolic processes in the mitochondrial inner membranes and mitochondrial protein complex (Supplementary Fig. S4f), pinpointing mitochondrial dysfunction at the onset of the disease.

These findings suggest that mitochondrial dysfunction and metabolic disorders manifesting in CMs decline even at the initial stage of cardiac. This underscores the necessity of investigating the mechanisms underlying FM in early disease pathogenesis.

### Phenotypic changes in CMs during FM progression

To determine the mechanisms driving functional changes in CMs at an early stage, we analyzed CMs with higher granularity. After dimension reduction and cell clustering, the CMs were further divided into 11 subclusters (Fig. 2b, c and Supplementary Fig. S5a and Table S2), each exhibiting distinct transcriptional characteristics (Supplementary Fig. S5b). To gain a deeper insight into the function of the CM subtypes, we defined 4 functional CM clusters (FC1–4) based on their transcriptome similarities, each of which expressed specific cell markers (Fig. 2d, e and Supplementary Fig. S5c, d and Table S3). Calculation of the cell proportion of each functional cluster (FC) indicated that the CMs of the normal heart mainly comprised FC1, followed by FC3, and a small portion of FC2. Drastic changes in cell composition were observed on days 4 and 7. A consistent reduction in the proportion of FC1 cells was observed during disease progression. FC2 proportion significantly increased on day 4, whereas that of FC4 increased significantly on day 7. The proportion of FC3 cells remained relatively stable (Fig. 2f). Changes in the proportion of FCs were validated using immunohistochemical staining (Fig. 2g). Functional enrichment analysis of individual FCs indicated that FC1 and FC3 displayed canonical CM properties, including several ATP metabolic processes (Fig. 2h). In addition, FC3 was enriched in the pathway of heart morphogenesis, indicating that this metabolic subtype primarily functions when the heart is in a diseased state (Fig. 2h). Therefore, we defined FC1 as “Healthy CMs” and FC3 as “Compensatory CMs”. In contrast, FC2 was mainly enriched in angiogenesis and defined as “Angiogenic CMs”. FC4 was predicted to exhibit inflammation-associated properties and was defined as “Inflammatory CMs” (Fig. 2h and Supplementary Fig. S5e). Accordingly, FC1 had a higher score for the tricarboxylic acid cycle, whereas FC2 and FC4 had higher angiogenesis and cytokine scores, respectively (Fig. 2i). The drastic decrease in healthy CMs and the increase in angiogenic CMs on day 4 in mice with FM may explain an early decrease in cardiac function.

Because of the remarkable proportional change in FCs during disease progression, we speculated that these FCs exhibit developmental relationships. Intriguingly, trajectory analysis indicated that FC1 could develop into FC2 and FC4, whereas FC3 was evenly distributed along the developmental trajectory (Fig. 2j, k and Supplementary Fig. S6a). Furthermore, the expression of the FC1 marker gene decreased, and that of the FC2 and FC4 marker genes increased along the developmental trajectory (Fig. 2l and Supplementary Fig. S6b and Table S4), whereas FC3 marker genes remained relatively stable (Supplementary Fig. S6c). This change in gene expression was consistent with our previous study, which reported bulk RNA-sequencing data from mice with FM^21^ (Supplementary Fig. S6d). Then, we examined the regulatory network underlying each FC using SCENIC and identified the specific TF regulons for each FC (Fig. 2m). Several regulons, such as *Klf4* and *Hand2*, which have been reported to be involved in phenotypic changes in CMs^14,22^, were decreased in FC1 and enriched in FC2 and FC4, indicating that these TFs may be involved in the phenotypic changes in CMs.

To determine the reason for this phenotypic change in CMs, we stimulated HL-1 and AC16 cells with CVB3. Although the relative level of CVB3 has elevated hundreds or even thousands of times in CMs after CVB3 infection (Supplementary Fig. S7a, b), the trajectory genes did not change in CMs for either RNA (Supplementary Fig. S7c, d) or protein levels (Supplementary Fig. S7e–h). These results indicated that phenotypic changes in CMs may be induced by an immune response rather than via direct stimulation by CVB3.

Collectively, these findings reveal that healthy CMs differentiate into angiogenic and inflammatory CMs and lose their original function during disease progression. Moreover, this process may be mediated by an inflammatory response rather than virus, highlighting the importance of elucidating the immune microenvironment in infected hearts.

### Cardiac neutrophils exhibit distinct developmental trajectory

**Fig. 3:**
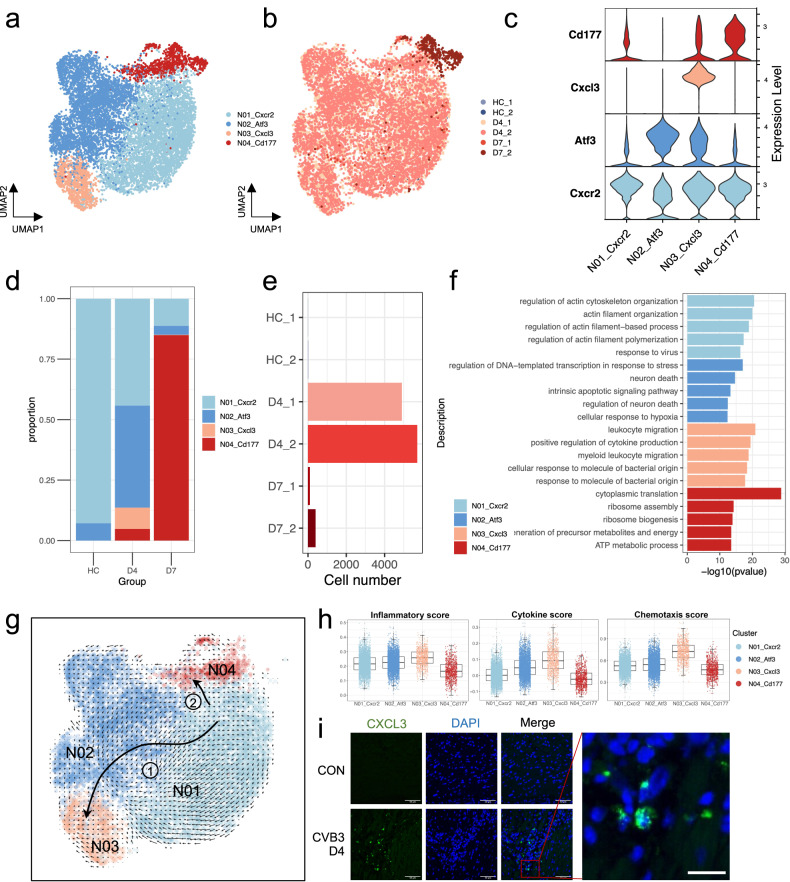
Characterization of cardiac neutrophils at different stages of FM. **a**, **b** UMAP embedding of neutrophils colored by manually annotated clusters (**a**) and samples (**b**). **c** Violin plot showing the expression levels of cell typing genes in each neutrophil subtype. **d** Cell proportion change of neutrophils at different time points. **e** Bar plot showing the total cell number of each sample. **f** GO analysis of specifically expressed genes in each neutrophil subtype. **g** UMAP plot of neutrophils’ developmental transition as revealed by RNA velocity. **h** Box plot of characteristic scores among different neutrophil subclusters. Scores were calculated by AddModuleScore function of the Seurat package. **i** Immunofluorescence staining of Cxcl3 in the heart sample of FM mice at day 4. Scale bars: 50 μm (20 μm in the zoomed image).

Neutrophils migrate from circulating blood to infected tissues in response to inflammatory stimuli and function as essential regulators of cardiovascular inflammation^23–25^. However, its role in myocarditis has been largely overlooked^26^. Based on our findings, cardiac contractile dysfunction was initiated on day 4 (Fig. 1a). Consistently, CMs on day 4 exhibited dramatic phenotypic changes, while neutrophils were the most abundant immune cells that infiltrated the myocardium (Fig. 1k). Therefore, we hypothesized that neutrophils play an important role in early immune response and are responsible for decreased cardiac function. To identify the characteristics of neutrophils, we performed unbiased graph-based clustering and identified 4 neutrophil subpopulations (Fig. 3a–c and Supplementary Fig. S8a, b and Table S5). The control group consisted of fewer neutrophils, most of which belonged to the N01 subtype (Fig. 3d, e and Supplementary Fig. S8a), which expressed Cxcr2 and was primarily involved in both the biogenesis process and the induced response to the virus (Fig. 3f). A small portion of N02 existed in the healthy heart, and the subpopulation expanded significantly by day 4. The function of N02 is mainly associated with apoptosis and cell death. N03 exclusively expressed Cxcl3, which appeared on day 4 and diminished on day 7. N03 was primarily enriched in the inflammatory pathways (Fig. 3f). N04 showed high expression of CD177, which became most abundant on day 7 (Fig. 3c, d).

To determine the relationship among neutrophil subgroups, we performed trajectory analyses. Both the velocity and slingshot analyses indicated a differentiation trajectory from N01 to N03 along N02 (Trajectory 1) and a trajectory from N01 to N04 (Trajectory 2) (Fig. 3g and Supplementary Fig. S8c). Because N02 and N03 expanded on day 4, whereas N04 expanded on day 7, Trajectory 1 may have been the predominant developmental process on day 4, whereas Trajectory 2 primarily existed on day 7. Along Trajectory 1, the inflammatory, cytokine, and chemotaxis scores increased, indicating an acquired pro-inflammatory phenotype following differentiation at the early stage (Fig. 3h). Immunofluorescent staining confirmed the presence of Cxcl3^+^ neutrophils in the heart on day 4 (Fig. 3i). A previous study showed that CD177^+^ neutrophils form neutrophil extracellular traps (NETs) during biliary atresia^27^. We examined whether these cells could form NETs in FM and observed increased NET formation on day 7 (Supplementary Fig. S8d). Our findings are consistent with those reported in a recent study, indicating that NETs play an important role in the inflammatory stage of viral myocarditis^28^.

Although monocytes were primarily observed in the heart on day 7, a small number of macrophages and monocytes were also observed in the inflamed heart on day 4 (Fig. 1k). Hence, we performed a second round of clustering analysis and identified five macrophage subclusters (Supplementary Fig. S9a–d). The M01 cluster was the most expanded subtype after infection (Supplementary Fig. S9e). This cluster highly expressed Ccr2 and represented pro-inflammatory macrophages that migrated from the blood (Supplementary Fig. S9f, g)^29,30^. Furthermore, this cluster showed strong interaction with CMs at day 7 (Supplementary Fig. S9h), which may mediate cardiac damage at this time point.

These results highlighted the heterogeneity of myeloid cells during disease progression. On day 4, neutrophils that migrated from the peripheral blood exhibited a developmental trajectory towards a more pro-inflammatory phenotype, which might have a distinct role in the collapse of cardiac function.

### Neutrophils undergo immune remodeling only after migrating into the heart

**Fig. 4:**
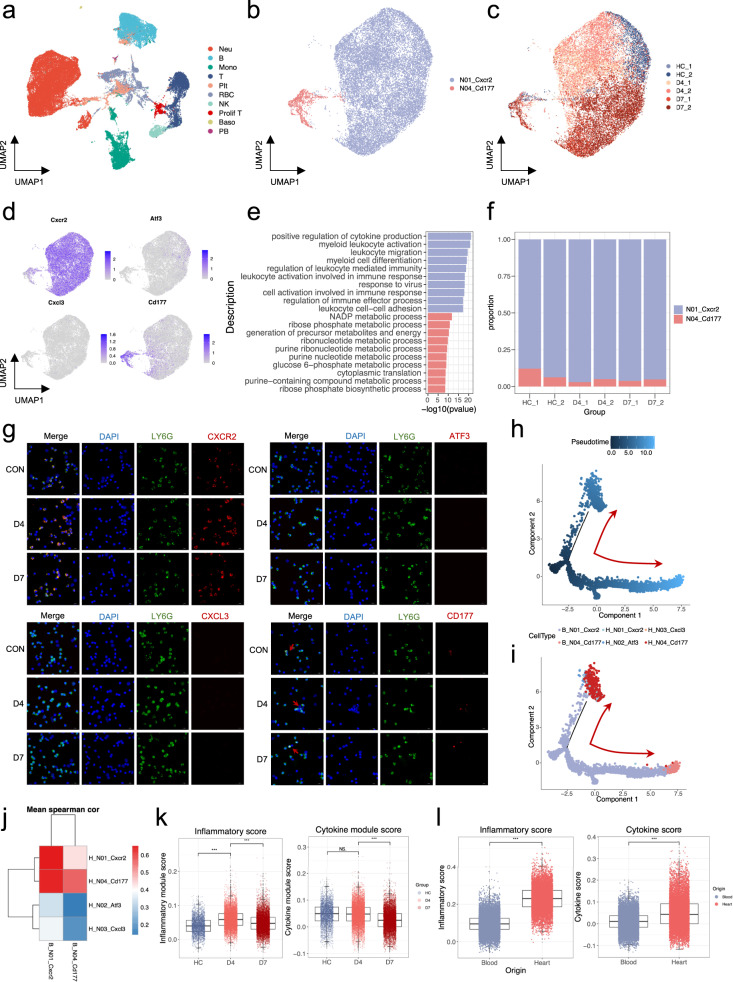
Characterization of peripheral neutrophils at different stages of FM. **a**–**c** UMAP embedding of peripheral blood immune cells colored by manually annotated clusters. UMAP embedding of peripheral neutrophils colored by manually annotated clusters (**b**) and samples (**c**). **d** FeaturePlot of marker genes of cardiac neutrophils. **e** GO analysis of specifically expressed genes in peripheral neutrophil clusters. **f** Cell proportion change of peripheral neutrophils at different time points. **g** Immunofluorescence staining of peripheral neutrophils at different time points. Scale bars: 20 μm. **h**, **i** Developmental trajectories of cardiac and peripheral neutrophils at day 7 colored by pseudotime (**h**) and cell types (**i**). **j** Spearman correlation of cardiac and peripheral neutrophil subtypes. **k** Box plot of characteristic scores of peripheral neutrophils at different time points. **l** Box plot of characteristic scores between cardiac and peripheral neutrophils.

As previously stated, only a few neutrophils are found in a healthy heart, and they mainly migrate from the peripheral blood. Elevated peripheral neutrophil level is a typical clinical manifestation of patients with FM. To better understand the origin and characteristics of neutrophils that infiltrate cardiac tissues, we performed scRNA-seq of the peripheral blood of mice with FM on days 0, 4, and 7 (Supplementary Fig. S10a–d) and identified 10 immune cell types (Fig. 4a and Supplementary Fig. S10e and Table S6). The proportion of peripheral neutrophils increased over time (Supplementary Fig. S10f), which was consistent with the trend observed in patients with FM.

Unbiased graph-based clustering identified two neutrophil subpopulations (Fig. 4b, c and Supplementary Fig. 11a, b and Table S7). A large proportion of peripheral neutrophils expressing Cxcr2, the marker gene of cardiac N01 cells, whereas a small proportion of cells expressing Cd177, the marker gene of cardiac N04 cells (Fig. 4d and Supplementary Fig. S11b). However, no marker genes of cardiac N02 or N03 cells were detected in peripheral neutrophils (Fig. 4d). Peripheral Cxcr2^+^ N01 was mainly enriched in the canonical neutrophil pathways, whereas peripheral Cd177^+^ N04 was mainly associated with metabolic processes (Fig. 4e). Both Cxcr2^+^ N01 and Cd177^+^ N04 were stable in peripheral blood during disease progression (Fig. 4f). Immunofluorescence staining showed similar results with no Atf3^+^ or Cxcl3^+^ neutrophils detected in the peripheral blood (Fig. 4g). These results indicated that the pro-inflammatory trajectory from N01 to N03 along N02 occurred only in the heart.

Cxcr2^+^ and Cd177^+^ neutrophils were present in both the peripheral blood and the local heart. To investigate the developmental program of blood and heart neutrophils, we integrated the blood and heart neutrophils and performed a trajectory analysis. The results showed that Cxcr2^+^ neutrophils in the peripheral blood could develop into peripheral CD177^+^ neutrophils; however, they could also develop into cardiac CD177^+^ neutrophils after migrating to the local heart (Fig. 4h, i). This indicates that cardiac and peripheral CD177^+^ neutrophils were formed by two different independent developmental trajectories. In addition, correlation analysis indicated high similarity between peripheral N01 and cardiac-infiltrating N01 and N04, whereas the correlation between peripheral and cardiac N04 was relatively low (Fig. 4j). Moreover, few similarities were observed between the peripheral neutrophils and cardiac N02 and N03 cells (Fig. 4j). These results indicated that the developmental trajectory from N01 to N03 only existed locally in the heart but not in the peripheral blood, whereas the trajectory from N01 to N04 was present in both the heart and blood.

To confirm this finding, we calculated the inflammatory and cytokine scores of peripheral neutrophils at different time points. Although the inflammatory score of peripheral neutrophils showed a slight increase on day 4, the cytokine scores showed no significant changes. Moreover, both the inflammatory and cytokine scores decreased on day 7 (Fig. 4k). However, upon entering the heart, the neutrophils showed profound activation, acquired higher inflammatory and chemotaxis scores, and exhibited higher cytokine formation abilities (Fig. 4l). In addition, the maturation and proliferation of the cells also increased (Supplementary Fig. S11c). A comparison of the DEG in blood and heart neutrophils on day 4 revealed that genes encoding inflammatory molecules and chemokine ligands, including *Il1a*, *Tnf*, *Cxcl2*, *Ccl3*, and *Ccl4*, were among the most highly upregulated genes (Supplementary Fig. S11d). These results indicated that the pro-inflammatory cytokines detected in the peripheral blood mainly originated locally from the heart rather than from the peripheral blood. Moreover, we examined the time point at which these molecules were released and discovered that pro-inflammatory cytokines such as Il1b, Il1rn, Tnf, Tnfaip3, Osm, and Mif were locally secreted by neutrophils in the heart rather than in the peripheral blood on day 4, and their expression levels decreased on day 7 (Supplementary Fig. S11e). These results demonstrated that day 4 is a crucial time point for disease progression, during which neutrophils play a significant role.

These results indicate that neutrophils acquire pro-inflammatory phenotypes and release a great number of cytokines only after migrating to the heart, rather than in peripheral blood at the early stage of the disease. Elevated peripheral neutrophil level may act as a reservoir for cardiac neutrophils.

### Neutrophils are the culprit of the inflammatory storm and promote the remodeling of cardiomyocytes at the onset of FM

**Fig. 5:**
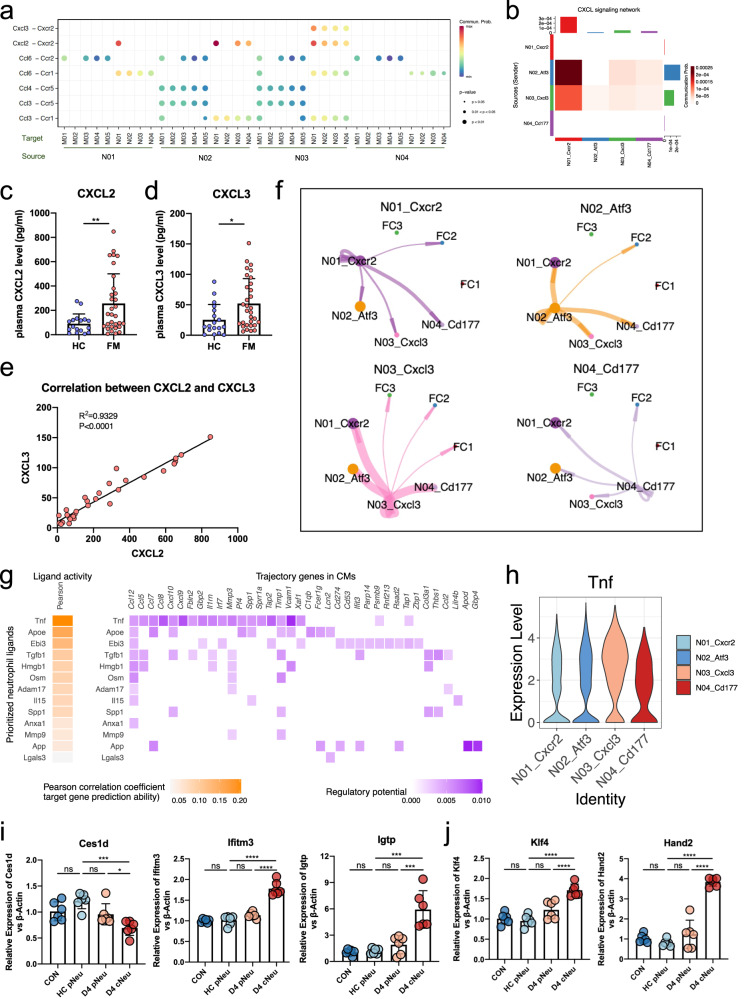
Functions of cardiac neutrophils. **a** Bubble plot showing the intercellular communication of chemotaxis among neutrophils and monocyte/macrophage subtypes, using CellChat working flow. **b** Heatmap showing the chemotaxis effects among cardiac neutrophil clusters through Cxcl–Cxcr. **c**, **d** The expression levels of Cxcl2 (**c**) and Cxcl3 (**d**) in the peripheral blood of patients with FM. **e** Correlation between the levels of Cxcl2 and Cxcl3 in the peripheral blood of patients with FM. **f** Interaction strength of ligand–receptor pairs between any pair of two cell populations among neutrophils and CM subclusters at day 4. **g** Heatmap showing the expression of ligand genes expressed in cardiac neutrophils at day 4 (left) and the regulated trajectory gene of those ligand expressions in CMs (right). **h** The expression of *Tnf* in each cardiac neutrophil cluster. **i**, **j** The relative expression of marker genes of FCs (**i**) and transcriptional factors (**j**) in cardiomyocytes of at different groups.

As the chemotactic ability of neutrophils increased when they differentiated from N01 to N03, we used CellChat to build cellular communication networks based on ligand and receptor expression in neutrophils and other immune cell subtypes to infer potential chemotaxis interactions. These results indicate active chemotactic interactions between various subtypes of neutrophils and macrophages (Fig. 5a). Neutrophils, particularly N02 and N03, expressed high levels of Cxcl2 and Cxcl3 to chemotaxis N01 via the Cxcl2/Cxcl3-Cxcr2 axis (Fig. 5b and Supplementary Fig. S12a). This indicates that the neutrophils congregating in the heart on day 4 were self-recruited. N01, derived from the peripheral blood, gradually developed into N03, which acquired a higher chemotactic ability and consistently attracted neutrophils from the peripheral blood, resulting in the rapid accumulation of neutrophils at the early stage of the disease. The levels of Cxcl2 and Cxcl3 were also elevated in the peripheral blood of patients with FM (Fig. 5c, d), and a high correlation was observed between the levels of Cxcl2 and Cxcl3 (Fig. 5e). In addition, neutrophils, particularly N03, acquired the ability to attract peripheral monocytes following differentiation by expressing high levels of Ccl3, Ccl4, and Ccl6 (Supplementary Fig. S12b, c), which may have caused monocyte accumulation in the heart at the later stage.

In addition to interacting with immune cells, neutrophils can directly interact with CMs. The CellChat analysis indicated that all four neutrophil clusters interacted with FC2 on day 4, whereas N03 also interacted with FC1 and FC3 (Fig. 5f). This suggests that N03 may be involved in phenotypic changes in CMs. Therefore, we calculated the modulatory effects of day 4 neutrophils on the elevated expression of CM trajectory genes, as shown in Fig. 2k. The results indicated that cytokines, particularly TNF, mediated the expression of trajectory genes in CMs (Fig. 5g), which was mainly released by N03 (Fig. 5h). To validate the pro-phenotypic effect of cardiac neutrophils on CMs at an early stage of the disease, we isolated neutrophils from the peripheral blood (pNeu) of FM mice on days 0 and 4, and cardiac-infiltrating neutrophils (cNeu) on day 4, and co-cultured them with CMs. The results indicated that CMs co-cultured with day 4 cNeu showed decreased *Ces1d* expression and increased *Ifitm3* and *Igtp* expression. This phenomenon was not observed in CMs co-cultured with both healthy and day 4 pNeu (Fig. 5i). These results highlighted that cardiac-infiltrating neutrophils can induce phenotypic changes in CMs. In addition, the transcription factors *Klf4* and *Hand2* were increased in CMs co-cultured with day 4 cNeu (Fig. 5j), indicating that they may have been involved in the differentiation of CMs induced by cardiac neutrophils at the onset of the disease.

In addition to heightened chemotactic capabilities, neutrophils also acquired high cytokine release ability (Fig. 3h). Among these cytokines, S100A8/A9 was highly expressed in neutrophils (Supplementary Fig. S12d) and remained high levels during disease progression (Supplementary Fig. S12e, f). S100 proteins are inflammatory indicators that interact with TLR4 via a positive feedback loop^31^. High *TLR4* expression was observed in both the N03 and M01 cells (Supplementary Fig. S12g, h), indicating that the pro-inflammatory N03 and M01 can be activated by S100A8/A9 released by neutrophils via TLR4 after reaching the heart. TLR4 activation in myeloid cells causes the production of reactive oxygen species, TNF-α, and IL-1β as well as oxidative stress and the activation of MAPKs and NF-kB pathway^32,33^. Thus, pro-inflammatory N03 and N01 may further boost the formation of an inflammatory storm after binding to neutrophil-released S100 proteins.

Collectively, after neutrophils migrated to the local heart and acquired pro-inflammatory phenotypes, they continuously self-recruited, directly induced remodeling of CMs and cardiac dysfunction, and triggered the firework of an “inflammatory storm” by attracting and activating pro-inflammatory monocytes at the early stage of the disease.

### Early blockade of Cxcr2 decreases cardiac immune infiltration and improves cardiac function

**Fig. 6:**
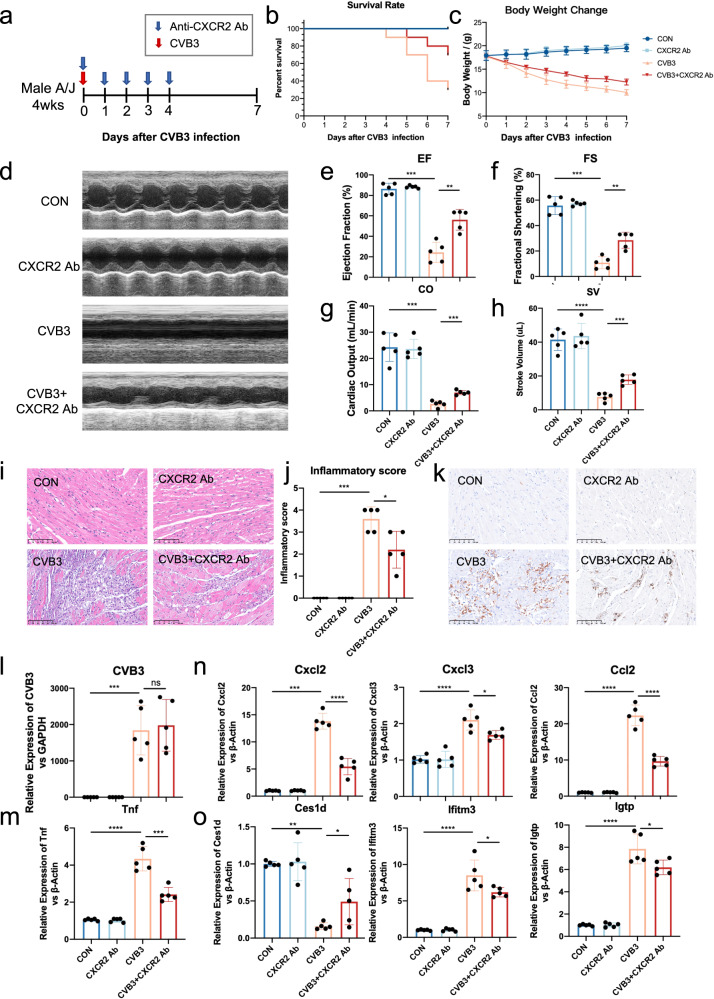
Blockade the self-recruiting loop of neutrophils at the early stage decreases cardiac immune infiltration and improves cardiac function. **a** Experimental layout of anti-Cxcr2 treatment. **b** Survival rate of FM mice during disease progression. **c** Body weight change of FM mice during disease progression. **d** Representative echocardiographic image of FM and Cxcr2 antibody-treated mice. **e**–**h** The ejection fraction (**e**), fraction shortening (**f**), cardiac output (**g**) and stroke volume (**h**) change of FM and Cxcr2 antibody-treated mice. **i, j** Representative images (**i**) and quantification (**j**) of cardiac inflammation in FM and Cxcr2 antibody-treated mice. Scale bars: 100 μm. **k** Representative Immunohistochemistry staining images of cardiac samples from FM mice at different time points. CD68 represents macrophages. Scale bars: 100 μm. **l** Cardiac CVB3 detection at different groups. **m**–**o** The relative expression of cytokines (**m**), chemokines (**n**), and marker genes of FCs (**o**) in cardiac samples of different groups.

To validate the detrimental role of cardiac-infiltrating neutrophils at the onset of FM, we investigated whether blocking the self-recruitment of neutrophils in the infected heart at an early stage by neutralizing Cxcr2 could improve cardiac dysfunction (Fig. 6a). Anti-Cxcr2 treatment significantly reduced the mortality rate of FM mice from > 60% to 30% (Fig. 6b). The general status of the mice also improved after treatment, as reflected by increased body weight (Fig. 6c). Cardiac function also significantly improved after treatment (Fig. 6d–h). Simultaneously, cardiac immune cell infiltration was significantly reduced (Fig. 6i, j). The infiltration of pro-inflammatory monocytes also decreased (Fig. 6k). However, the copy number of CVB3 in the heart did not change after the treatment (Fig. 6l). This indicates that compared to direct CVB3-induced damage, an over-activated immune response might contribute more to heart damage. The expression of common cytokines (Fig. 6m) and chemokines (Fig. 6n) in the heart also decreased, indicating restriction of the inflammatory storm. Moreover, we discovered that the phenotypic transition of CMs was partially relieved by Cxcr2 neutralizing antibody treatment (Fig. 6o). In addition, the combined blockade of Cxcl2 and Cxcl3 resulted in similar results to the Cxcr2 blockade (Supplementary Fig. S13a), which significantly reduced the death rate of FM mice and improved their cardiac function (Supplementary Fig. S13b–g).

Therefore, blocking the self-recruitment of neutrophils from the blood to the infected heart successfully reduces the death rate and severity of cardiac deterioration in mice with FM. Furthermore, the treatment decreases cardiac inflammatory infiltration and cytokine release and relieves the phenotypic transition of CMs, highlighting its clinical significance.

## Discussion

Here, we present the comprehensive chronemics of a single-cell resolution catalog of both CMs and immune cells in the heart and peripheral blood associated with FM. This study provides detailed information on the pivotal and detrimental effects of self-recruiting neutrophils by identifying novel cell subtypes and altered pathways, analyzing cellular interactions, and highlighting the developmental trajectory of immune cells. Furthermore, the results identified a differentiation trajectory of cardiac neutrophils towards a pro-inflammatory phenotype, which led to acute cardiac collapse during FM (Supplementary Fig. S14).

Although we were unable to describe each subtype and its biological functions in detail, the key observations are crucial. First, we discover that cardiac function is reduced on day 4, followed by a rapid deterioration of cardiac function. The primary cause of this phenomenon is that transcriptional changes in CMs are drastic between day 0 and day 4. Furthermore, transcriptional changes in CMs are primarily attributable to phenotypic changes during the progression of FM from functionally normal to pro-angiogenic and pro-inflammatory CMs. Fatty acid-based energy metabolism contributes up to 70% of cardiac ATP^34^. Loss of healthy metabolic CMs may explain the deterioration of cardiac function in patients with FM. The direct cytopathic effect of viral replication in cardiac cells is one of the main causes of cardiac damage in myocarditis^6,35^. However, we discovered that direct stimulation of CMs in vitro with CVB3 did not result in the same phenotypic changes as those observed in vivo, highlighting the importance of inflammation-mediated damage to CMs.

Second, we observe marked neutrophil accumulation in the heart 4 days after CVB3 infection, which is consistent with the well-known nature of rapid chemotaxis and migration to acutely injured tissues^36,37^. CVB3 can directly activate neutrophils via endosomal TLR8 and trigger NF-kB activation^38^. However, because immune cells are highly heterogeneous^39^, the diversity of neutrophils and their roles in FM require further investigation. The present study discovered that among cardiac-infiltrating neutrophils, the Cxcr2^+^ N01 cluster is the first to migrate from the blood to the heart and then develop into Atf3^+^ N02 and Cxcl3^+^ N03 clusters during the early cardiac dysfunction stage. Neutrophils gradually acquire the ability for increased cytokine production and chemotaxis when traveling along a differentiation trajectory. In addition, the Cxcr2^+^ N01 cluster also develops into the NETs-forming Cd177^+^ N04 cluster at the acute inflammatory stage (day 7), in agreement with a previous study showing that the ablation of NETs in mice with viral myocarditis at the acute inflammatory stage reduced tissue damage^38^. Moreover, the development trajectory from N01 to N03 primarily occurs in the heart rather than in the peripheral blood. The inflammatory and cytokine scores of peripheral myeloid cells showed no significant changes, emphasizing the importance of the local tissue milieu in immune cell fate^40^. Following the trajectory from N01 to N03, neutrophils acquire profound pro-inflammatory characteristics and release numerous cytokines, particularly on day 4. Elevated cytokine levels may aid cytokine-targeted treatments. High Il1b expression may explain the case reports of the effectiveness of anakinra treatment in patients with FM^41–43^. Notably, the levels of these cytokines are elevated during the early stage and decreased during the acute inflammatory stage, highlighting the importance of early diagnosis and treatment.

Third, we discover that the transient developmental trajectory from N01 to N03 on day 4 plays a significant role in cardiac dysfunction and induced inflammatory storms. Cxcr2^+^ N01 cells migrating from peripheral blood show low chemotactic ability. However, when these cells develop into Cxcl3^+^ N03 cells, they express high levels of chemokine ligands and continuously attract peripheral neutrophils to the heart. A positive feedback loop results in neutrophil accumulation. Moreover, well-developed neutrophils attract a large number of monocytes by releasing chemokines from the CCR family, resulting in monocyte accumulation later on day 7. Moreover, after migrating to the heart, neutrophils release numerous damage-associated molecular patterns (DAMPs), particularly S100A8/A9. Increased S100A8/A9 levels are recognized by DAMP-sensing receptors and their binding initiates cascades of inflammatory responses^44^. This study revealed that TLR4, the receptor for S100A8/A9, is expressed in both pro-inflammatory N03 and M01 cells. Elevated S100A8/S100A9 levels govern cytokine storm in severe coronavirus disease 2019 and correlate with poor prognosis^45,46^. Additionally, S100a8/a9 signaling can induce mitochondrial dysfunction and CM death in response to ischemic/reperfusion injury^47^ and could serve as a therapeutic target in inflammatory cardiomyopathies^48^. Therefore, S100A8/A9 expression in cardiac neutrophils may aggravate FM symptoms. Moreover, cytokines expressed by activated neutrophils, especially Cxcl3^+^ N03, can promote phenotypic changes in CMs, resulting in cardiac dysfunction. Thus, once neutrophils migrate to the heart, the joint effects of the aforementioned phenomena mediate an inflammatory storm and cardiac dysfunction. Therefore, disruption of the positive feedback loop of neutrophil self-recruitment may be a potential therapeutic strategy for treating cardiac immune responses.

Finally, we prove that early blockage of neutrophil migration from the peripheral blood significantly reduces mortality in mice with FM and attenuates cardiac dysfunction, with a reduction in both cardiac inflammation and cytokine release. Ccl2–Ccr2, a ligand–receptor pair that attracts monocytes, was reported to be an important therapeutic target in myocarditis^18^. This study discovers that neutrophils migrate earlier than monocytes, play a key role in early cardiac functional collapse, and attract monocytes that infiltrate the heart during acute inflammation. Thus, the early blockage of self-recruited neutrophils may serve as a significant therapeutic target for FM.

Our study has several limitations, one of which is the lack of information regarding cardiac endothelial cells and fibroblasts. In addition, particular cell subsets and conclusions derived from cell-state transition analyses require further experimental validation. These findings may aid in the development of novel therapeutic approaches for FM.

Collectively, our study provides unique insights into the physiopathology of CMs and immune cells in FM and elucidates previously unknown functional pathways and cell developmental trajectories. This provides a critical resource and important insight into the pathogenesis of FM and helps to fully elucidate the developmental process of the disease. Furthermore, these results may aid in the development of effective therapeutics.

## Materials and methods

### Animal studies

This study was approved by the Institutional Animal Research Committee of Tongji Medical College. All animal experimental protocols comply with the Guide for the Care and Use of Laboratory Animals, published by the National Institutes of Health. 6-week-old male A/J mice were obtained from GemPharmatech (Nanjing, China). A/J mice in the CVB3 group were intraperitoneally injected with 1 × 10^4^ plaque-forming units of CVB3 as described previously^21^. Phosphate-buffered saline (PBS) was used as a control. The mice were observed daily, and body weight changes and survival events were recorded until death or at the designated time points. All animals were anaesthetized by intraperitoneal injection of a mixture of xylazine (5 mg/kg) and ketamine (80 mg/kg) before euthanasia. Echocardiography was performed to record cardiac functional changes using a Vevo1100® high-resolution imaging system with a 30-MHz high-frequency scan head (Visual Sonics, Canada). In the antibody treatment group, the Cxcr2 inhibitor SB225002 (Tocris) was administered intraperitoneally at a dose of 10 mg/kg daily from days 0 to day 4. The anti-Cxcl2 antibody (Cat#: ARG56661, Arigobio) and anti-Cxcl3 (Cat#: ARG56693, Arigobio) antibodies were administered intraperitoneally from days 0 to day 4. The samples were collected after euthanasia.

### Cell lines

The human myocardial cell line AC16 was obtained from the American Type Culture Collection and cultured in Dulbecco’s Modified Eagle’s Medium (Gibco, Grand Island, NY, USA) supplemented with 10% fetal bovine serum (FBS, Gibco). The murine cardiac muscle cell line HL-1, a kind gift from Professor Claycomb, was maintained in Claycomb medium (Sigma-Aldrich, St. Louis, MO, USA) with 10% FBS supplemented with 100 μM norepinephrine and 4 mM L-glutamine. Cells were seeded in 6-well plates and treated with CVB3 (1 × 10^4^ plaque-forming units) at 70%–80% confluence, as described previously^21^. The cells were harvested after stimulation for 24 h.

### Human samples

Autopsy samples from FM patients were acquired from the Department of Forensic Medicine, Tongji Medical College, Huazhong University of Science and Technology. All patients and healthy persons were recruited at the Tongji Hospital, Wuhan, China, between April 2017 and March 2021. The study was authorized by the ethical review boards of Tongji Hospital and Tongji Medical College (ID: TJ-C20160202) and complied with the Declaration of Helsinki standards. Written informed consent was obtained from all the participants. FM was diagnosed based on the 2009 International Consensus Group on Cardiovascular Magnetic Resonance in Myocarditis statement, position statements from the 2013 European Society of Cardiology, and the 2017 Chinese Society of Cardiology expert consensus statement^49–51^.

### Histological analyses

The heart tissue samples were fixed in 4% paraformaldehyde and embedded in paraffin. Tissue sections with a thickness of 4 μm were then subjected to haematoxylin-eosin and immunohistochemical or immunofluorescence staining. The inflammatory score was calculated as previously described^52^. Antibodies used in this study are listed in Supplementary Table S8.

### Protein extraction and western blotting

BCA method was used to determine the protein concentrations. The cell lysate was resolved by SDS-PAGE, transferred to a nitrocellulose membrane, and blocked with 5% non-fat dried milk in TBS-T. The membrane was first incubated with the designated primary antibody overnight at 4 °C, and then incubated with a peroxidase-conjugated secondary antibody for 2 h at room temperature. The ECL system was used for the final development (Beyotime Institute of Biotechnology, Nanjing, China). Antibodies used in this study are listed in Supplementary Table S8. Quantification and processing of the western blot results were performed using ImageJ software (National Institutes of Health).

### RNA isolation and qPCR

Total RNA extracted from heart tissue was transcribed into cDNA and analyzed using real-time qPCR. cDNA was amplified using the Taq Pro Universal SYBR qPCR Master Mix (Cat. Q712-02; Vazyme) and specific primers using a 7900HT Fast Real-Time PCR system (Life Technologies, Carlsbad, CA, USA). Gene expression was normalized to that of glycer-aldehyde-3-phosphate dehydrogenase and β-actin using the comparative CT method. Primer sequences used in this study are listed in Supplementary Table S9.

### Enzyme-linked immunosorbent assay

The plasma levels of Cxcl2 and Cxcl3 were measured using a standard enzyme-linked immunosorbent assay kit (Cat#: EHC165b.96, QuantiCyto; Cat#: EK1265, MULTI SCIENCES). All measurements were performed following the manufacturer’s instructions.

### Co-culture experiment

To isolate neutrophils, blood was collected from the angular veins of mice with FM on days 0 and 4. Hearts were collected from mice with FM on day 4 and cut in isotonic PBS (supplemented with 0.5% BSA). Hearts were digested with collagenase 4 (cat. Ls004188) for 45 min at 37 °C. After filtration and red blood cell lysis, peripheral and cardiac neutrophils were isolated using the MojoSort^TM^ Mouse Neutrophil Isolation Kit (Cat #480058, BioLegend) and co-cultured with mouse CMs (HL-1 cell line) for 12 h.

### Cell isolation and single-cell suspension preparation

Two platforms were used to elucidate the transcriptional changes and cellular landscape of CMs and cardiac immune cells. The hearts collected from mice of different groups were subjected to Langendorff retrograde perfusion by sequential calcium-free solution and enzymatic digestion (0.03 g BSA and 0.03 g collagenase dissolved in 45 mL of Ca^2+^-free solution). The tissue was blown, filtered, and centrifuged (100× *g* for 3 min) to obtain a single-cell suspension of CMs. To obtain additional immune cells, five hearts from each group were cut in isotonic PBS (supplemented with 0.5% BSA) and digested with collagenase 4 (Worthington, Cat#: Ls004188) for 45 min at 37 °C. After filtering and red blood cell lysis, the cell suspension was labeled with 1 µL/100 uL CD45-BV421 antibody (BioLegend, Cat#: 103134) and propidium iodide solution (BioLegend, Cat#: 421301) and subjected to fluorescence-activated cell sorting to acquire PI^-^CD45^+^ live immune cells. Peripheral immune cells were obtained at different time points by collecting blood from the angular veins of FM mice. The cells were labeled with 1 µL/100 µL of CD45-BV421 antibody (BioLegend, Cat#: 103134) and propidium iodide solution (BioLegend, Cat# 421301) and subjected to fluorescence-activated cell sorting after red blood cell lysis.

### scRNA-seq library preparation and sequencing

A single-cell sequencing library of CMs was generated using the iCell8 platform (Takara) as previously described^53^. Single-cell capture and library construction of cardiac immune cells and peripheral blood were performed using Chromium Next GEM Single Cell V(D)J Reagent Kits v1, following the manufacturer’s instructions (SeekGene, Beijing, China). Sequencing was performed on a NovaSeq platform (Illumina, San Diego, USA) to generate 150 bp paired-end reads.

### scRNA-seq data processing

Raw reads of CMs were processed using the Perl pipeline script supplied by WaferGen and filtered to remove low-quality cells, as previously described^53^. The cells were clustered using the Seurat package. Single-cell transcriptome data of immune cells were analyzed using the Cell Ranger Software Suite^54^ to perform alignment, filtering, barcode separation, and unique molecular identifier (UMI) counting using default parameters. Feature barcode matrices of each sample were generated for downstream analysis. Quality control was applied to the cells based on four metrics: the total UMI count, number of detected genes, proportion of mitochondrial gene counts per cell, and proportion of hemoglobin gene counts per cell. Specifically, cells that (1) expressed < 500 or > 4000 genes, (2) exhibited UMI counts < 1000 or > 15,000, (3) contained > 10% mitochondrial genes, and (4) contained > 1% hemoglobin genes were filtered. Additionally, we used DoubletFinder^55^ to identify and remove potential doublets in each sample with an expected doublet rate of (cell number) × 8 × 1e–6, and default parameters were used otherwise. After quality control and doublet removal, single cells with high qualities were remained for further analysis. The remaining cells were analyzed using the Seurat package to generate gene expression matrices using the NormalizeData and ScaleData functions^56,57^.

### Batch-effect correction and cell subset annotations

The Harmony algorithm was used to integrate the cells from different samples into a shared space for unsupervised clustering^58^. The genes used for the harmony algorithm were calculated using the SCTransform function in the Seurat package^59^. Next, we calculated a principal component analysis matrix using these variable genes and inputted this matrix into the RunHarmony function of the Harmony package. After batch-effect correction, the resulting batch-corrected matrix was used for unsupervised clustering using the RunUMAP function. Marker genes in each cluster were identified using the FindClusters function of the Seurat package.

The first round of clustering (resolution = 0.3) identified 5 cell types in CM samples and 11 cell types in immune cell samples, according to their specific cell markers. To identify subclusters within the major cell types, we performed a second round of neutrophil and macrophage clustering. The procedure for the second round of clustering was the same as that of the first round, and cell cluster annotation was based on the expression of canonical cell-typing markers. During this process, cells expressing two sets of well-studied markers of major cell types were recognized as doublets and removed.

### Differential expression and GO enrichment analysis

Differential expression analysis was performed using the FindMarkers function of the Seurat package with the default parameters. *p*_val_adj < 0.05 and |avg_log2FC| >  0.25 were used to define significant DEGs. Based on these DEGs, enriched GO terms were acquired using the enrichGO function of the clusterProfiler package^60^ following the default parameters. The annotation Dbi R package “org.Mm.eg.db” was used to map gene identifiers.

### Cellular interaction

NicheNet^61^ was used to infer cell-to-cell interactions between neutrophils and CMs using the default parameters. Cell–cell interactions based on the expression of known ligand–receptor pairs between monocytes and CMs and between neutrophils and CMs were inferred using the CellChat (version 1.0.0) R package^62^ (FC4 is not included in this calculation due to less than 10 FC4 cells existed in day 4). We used default settings to predict the major signaling interactions between cells and the coordination of various functions between these cells and signals. Briefly, we followed the workflow recommended in CellChat, loaded the normalized counts into CellChat, and applied the preprocessing functions identifyOverExpressedGenes, identifyOverExpressedInteractions, and projectData with default parameter sets. For the analysis of ligand–receptor interactions, the functions computeCommunProb, computeCommunProbPathway, and aggregateNet were applied using default parameters.

### Pseudotime analysis

Pseudotime analyses were performed on FC1, FC2, FC3 and FC4 from the CM data and on cardiac and peripheral neutrophil subtypes using the Monocle2 R package^63,64^. Gene order was performed using a cutoff for expression in at least ten cells and a combination of inter-cluster differential expression and dispersion, with a *q*-value cutoff of < 0.01. The structure of the trajectory was plotted in two-dimensional space using the DDRTree dimensionality reduction algorithm, and the cells were ordered in pseudotime.

### RNA velocity and slingshot analysis

Both RNA velocity^65^ and slingshot analyses^66^ have been used to infer the developmental trends of neutrophils. For RNA velocity analysis, a loom file representing the spliced and unspliced information of each sample was constructed using the Velocyto (version 0.17.17) Python package based on the bam files from the Cell Ranger toolkit. After merging all loom files, we separated the cell clusters of interest. Data were normalized and scaled with the “SCTransform” function with the top 2000 highly variable genes. Clustering information was imported from the Seurat results, and the UMAP coordinates were rerun using “RunUMAP” for visualization. RNA velocity values were calculated by the “RunVelocity” function of the Seurat-Wrappers package, and visualization was performed by the “show.velocity.on.embedding.cor” function with the default parameter. For slingshot analysis, the slingshot function of the Slingshot^66^ (v1.6.0) R package was used with default settings, and UMAP embeddings were used to plot the trajectory for each cell type.

### Module score calculation

Module scores were evaluated using the AddModuleScore function in the Seurat package^57^. All genes used to calculate gene scores are listed in Supplementary Table S10.

### Regulatory network inference

A single-cell regulatory network for CMs was constructed with SCENIC^67^. Specifically, GENIE3 was used to infer gene regulatory networks from the raw count data. Potential direct-binding targets (regulons) were selected based on a DNA motif analysis using RcisTarget. Finally, the gene regulatory network activity of each cell type was identified using AUCell. Regular activity was averaged to determine the regulators of the CM clusters. A regulon-group- or regulon-cell-type heatmap was generated using the R pheatmap package.

### Quantification and statistical analysis

Statistical analyses were performed using the R software (version 3.6.1) and GraphPad Prism V.4 (GraphPad Software). Wilcoxon rank-sum test and Student’s *t*-test were used in this study. *P < 0.05, **P < 0.01, and ***P < 0.001 were considered statistically significant. Survival analysis was performed using the log-rank test.

### Supplementary information

Supplementary Figures

Supplementary Tables S1–S10

## Data Availability

The accession number for the scRNA-seq data reported in this study is NGDC database subPRO014661. A secure token was created to allow the review of record data while they remain in private status.
