# Supplementary material for: Self-recruited neutrophils trigger over-activated innate immune response and phenotypic change of cardiomyocytes in fulminant viral myocarditis
Source: Cell Discov. 2023 Oct 10;9:103. doi: 10.1038/s41421-023-00593-5 (PMC10564723; doi:10.1038/s41421-023-00593-5)
Supplement: Supplementary file 1 — Supplementary Figures [file 41421_2023_593_MOESM1_ESM.pdf]

Supplementary Fig. S1

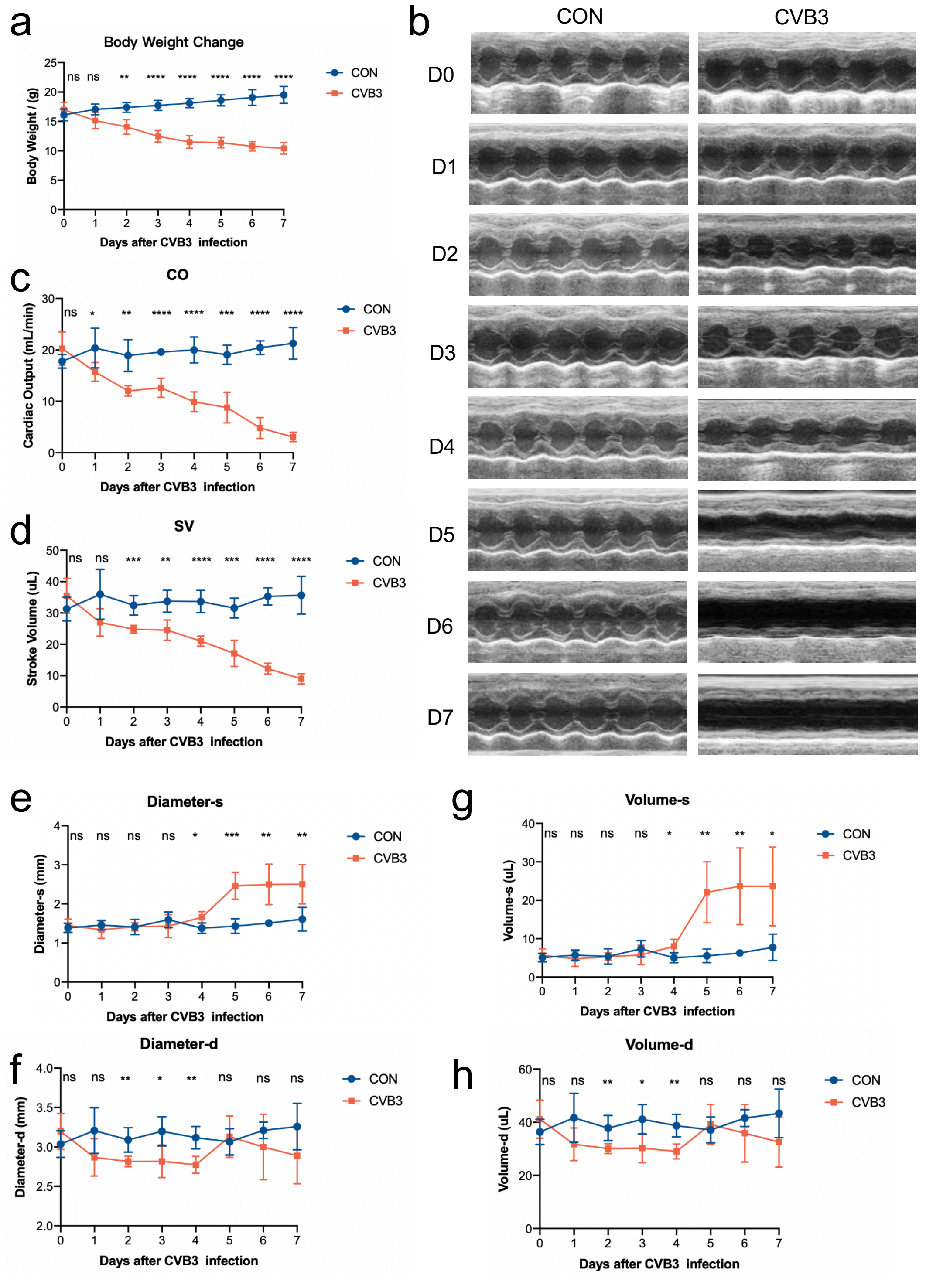

Supplementary Fig. S1 Body weight change and cardiac function of FM mice. a Body weight change of FM mice during disease progression. **b** Representative echocardiographic images of FM mice during disease progression. Cardiac output (**c**), S stroke volume (**d**), Diameter-s (**e**), Diameter-d (**f**), Volume-s (**g**), Volume-d (**h**) changes during disease progression.

Supplementary Fig. S2

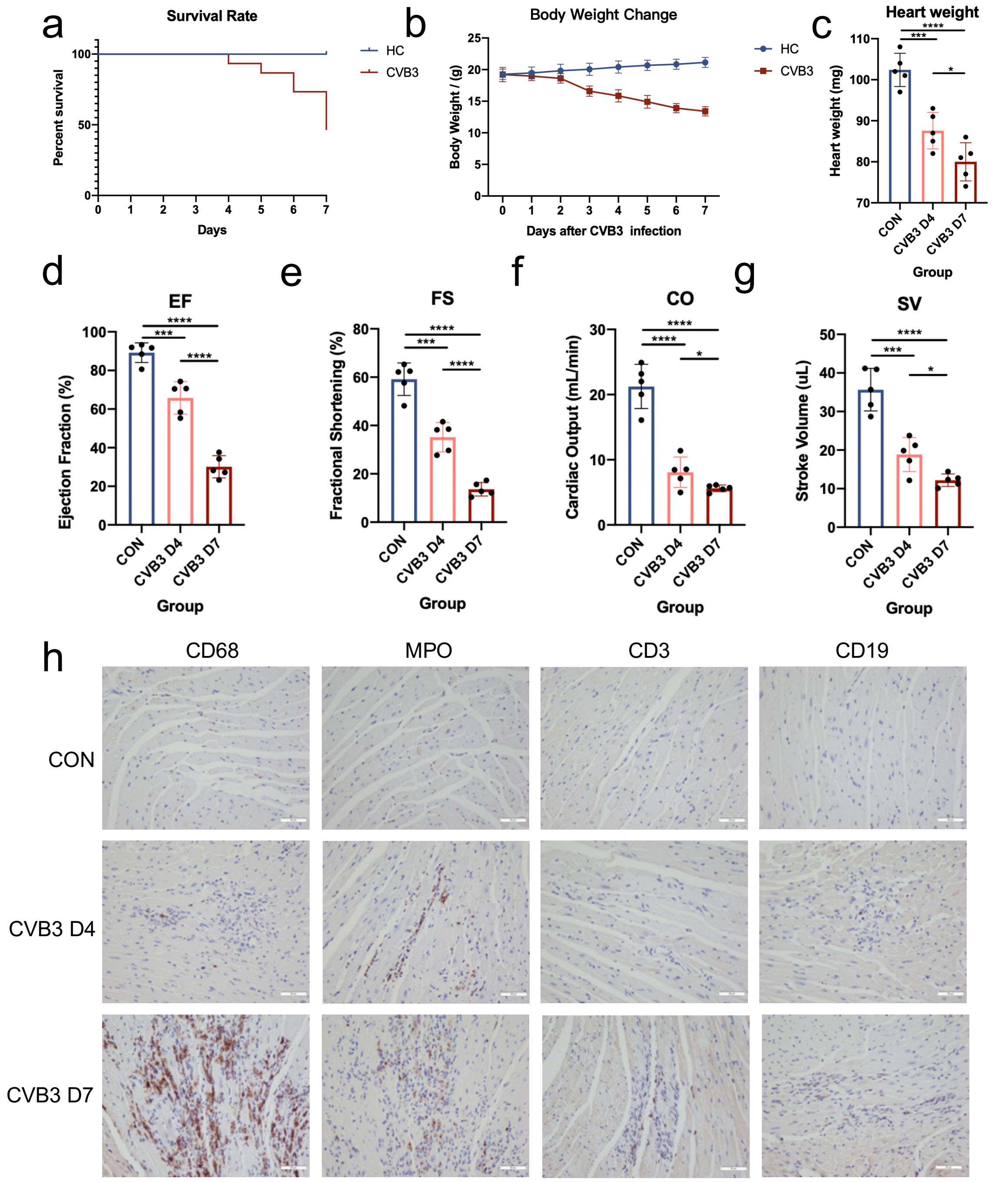

Supplementary Fig. S2 Survival rate, cardiac function, and cardiac immune infiltration of FM mice. Survival rate (**a**), body weight (**b**) and heart weight (**c**) change of FM mice during disease progression. Ejection fraction (**d**), fraction shortening (**e**), cardiac output (**f**) and stroke volume (**g**) changes during disease progression. **h** Representative Immunobiological staining images of cardiac samples from FM mice at different time points. CD68, MPO, CD3 and CD19 represent macrophages, neutrophils, T cells and B cells respectively (Scale bar: 50μm).

Supplementary Fig. S3

a

Sample	Cell number	UMI count	Gene count
HC_1	430	504092	3526
HC_2	550	503689	3216
D4_1	638	271648	2714
D4_2	307	266416	2580
D7_1	1052	264730	2497
D7_2	1091	302635	3458

b

Sample	Cell number	UMI count	Gene count
HC_1	11410	3273	1401
HC_2	7214	5085	1874
D4_1	8180	3110	1084
D4_2	7776	3031	961
D7_1	10154	4661	1812
D7_2	7977	4735	1904

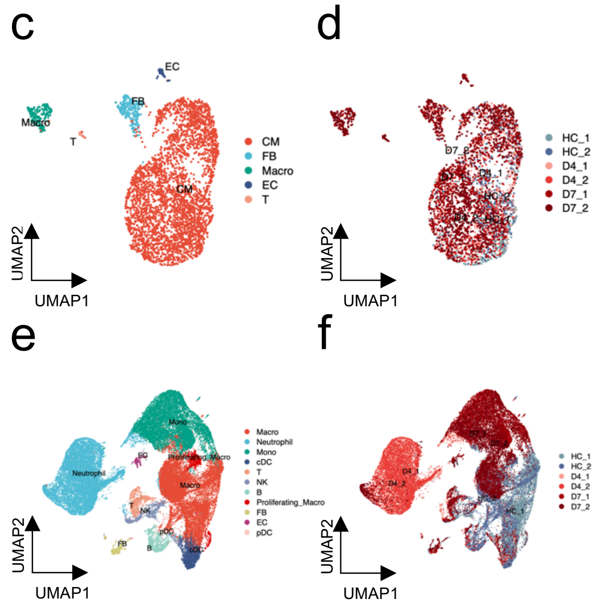

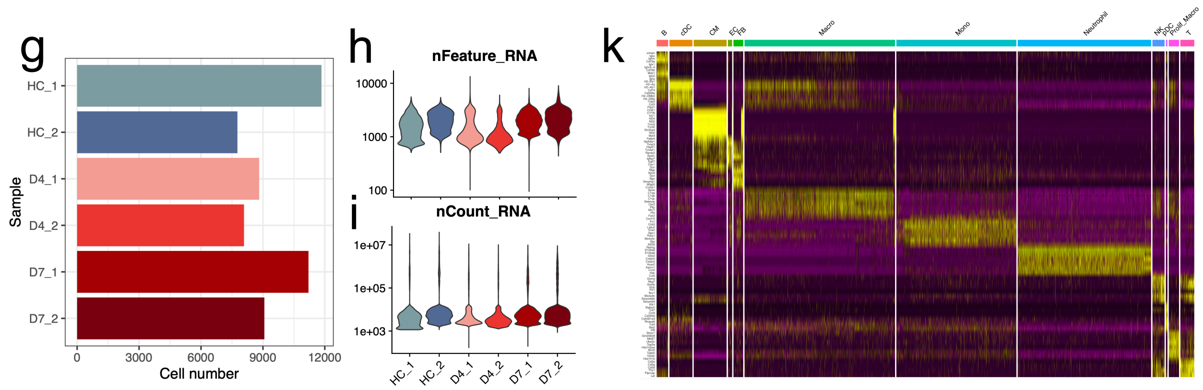

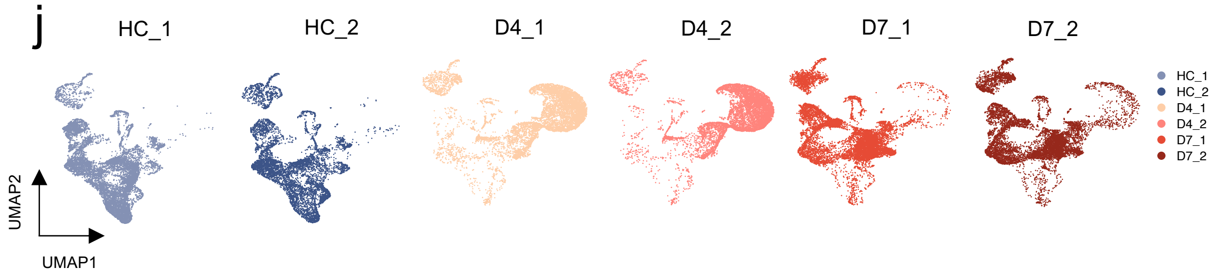

l

	B	cDC	CM	EC	FB	Macro	Mono	Neutrophil	NK	pDC	Proliferat-ing_Macro	T
HC_1	128	1498	413	55	107	7807	368	22	504	57	468	413
HC_2	860	675	514	68	163	4176	327	43	385	41	259	253
D4_1	89	91	588	43	97	337	523	6723	119	26	17	165
D4_2	73	50	286	33	48	148	298	6929	66	15	5	132
D7_1	50	103	892	137	305	1396	7442	200	151	25	172	333
D7_2	65	81	859	140	327	2465	4078	539	84	15	209	206

Supplementary Fig. S3 Preprocessing of scRNAseq data. Cell number, UMI count and gene count of each cardiomyocyte samples (**a**) and immune cell samples (**b**). UMAP embedding of CMs from all profiled samples colored

by cell types (**c**) and samples (**d**). UMAP embedding of immune cells from all profiled samples colored by cell types (**e**) and samples (**f**). **g** Bar plot showed the total cell number of each sample. Violine plot of log10 transformed nFeature_RNA (**h**) and nCount_RNA (**i**) of each sample. **j** UMAP embedding of all cells split by samples. **k** Heatmap showing top 10 DEGs among all cell types. **l** Numbers of each cell type in different samples.

Supplementary Fig. S4

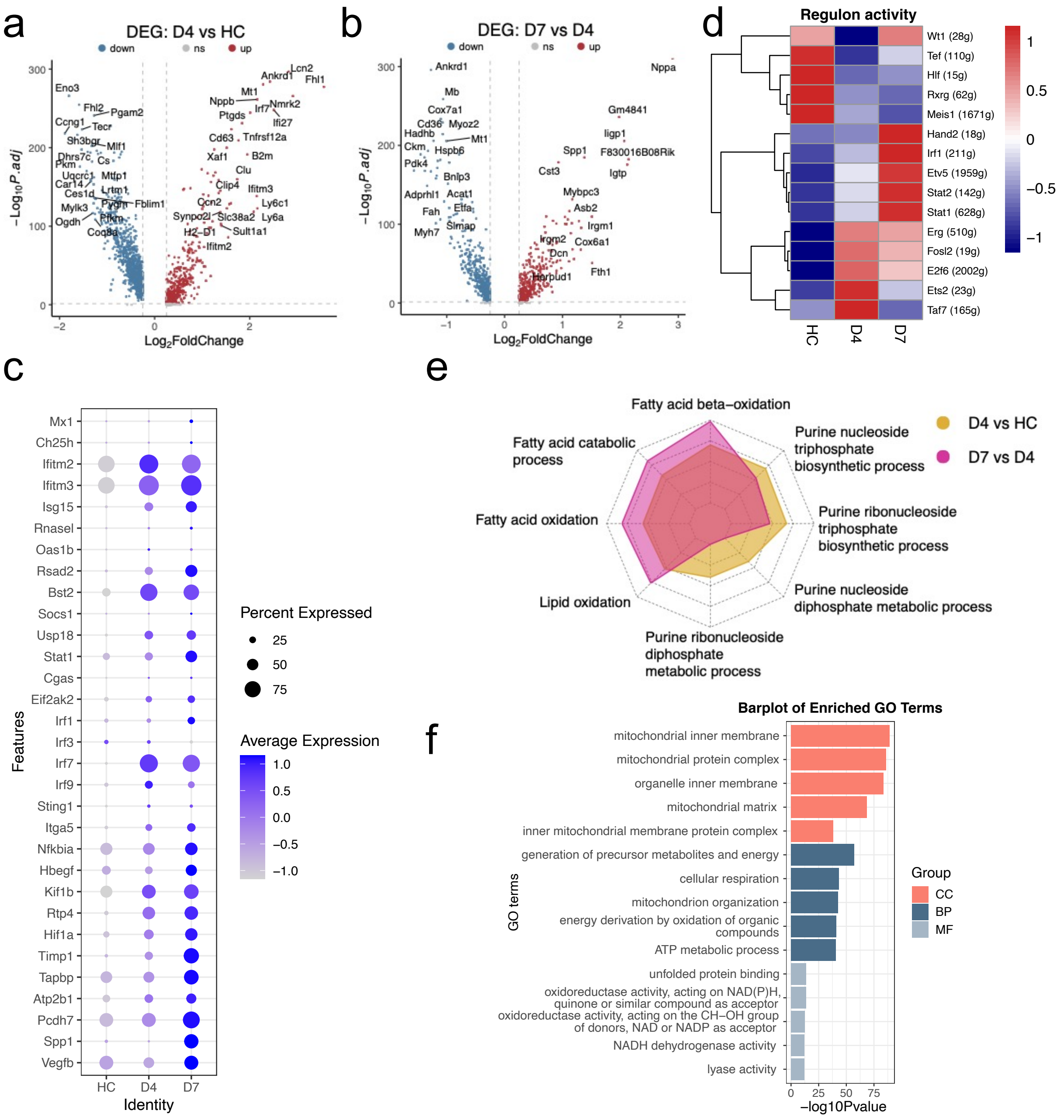

Supplementary Fig. S4 Differential analysis and gene enrichment analysis of CMs. Volcano plot showed the regulated genes between CMs of D4 and HC (**a**), D7 and D4 (**b**). **c** Dot plot showed the expression levels of IFN genes and inflammatory genes in each sample. Dot color represented the gene expression level, and dot size represented the percentage of cells expressing the respective gene. **d** Heatmap of regulon activity in CM at different time points. **e** Radar plot shows GO enrichment of decreased genes at different time point. **f** Top down regulated pathways between CMs of D4 and HC.

Supplementary Fig. S5

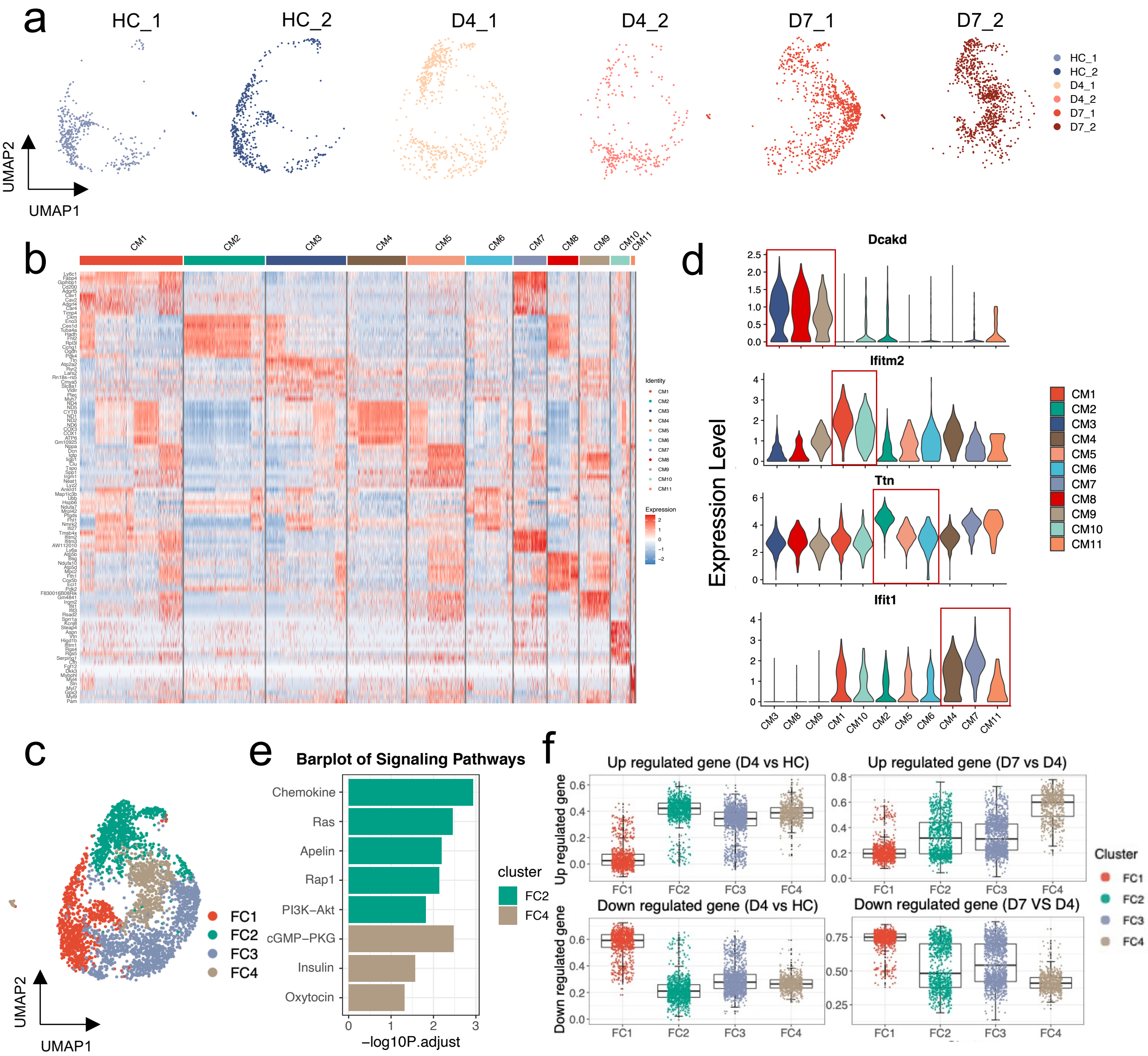

Supplementary Fig. S5 Characteristics of functional clusters of cardiomyocytes. a UMAP embedding of CMs split by samples. **b** Heatmap showing top 10 DEGs among 11 CMs clusters. **c** UMAP embedding of all cells colored by FCs. **d** Expression of marker genes for each FC. **e** Top regulated signaling pathways between FC2 and FC4 calculated by KEGG. **f** The expression level of the up and down regulated genes of cardiomyocytes at different time points in each FCs.

Supplementary Fig. S6

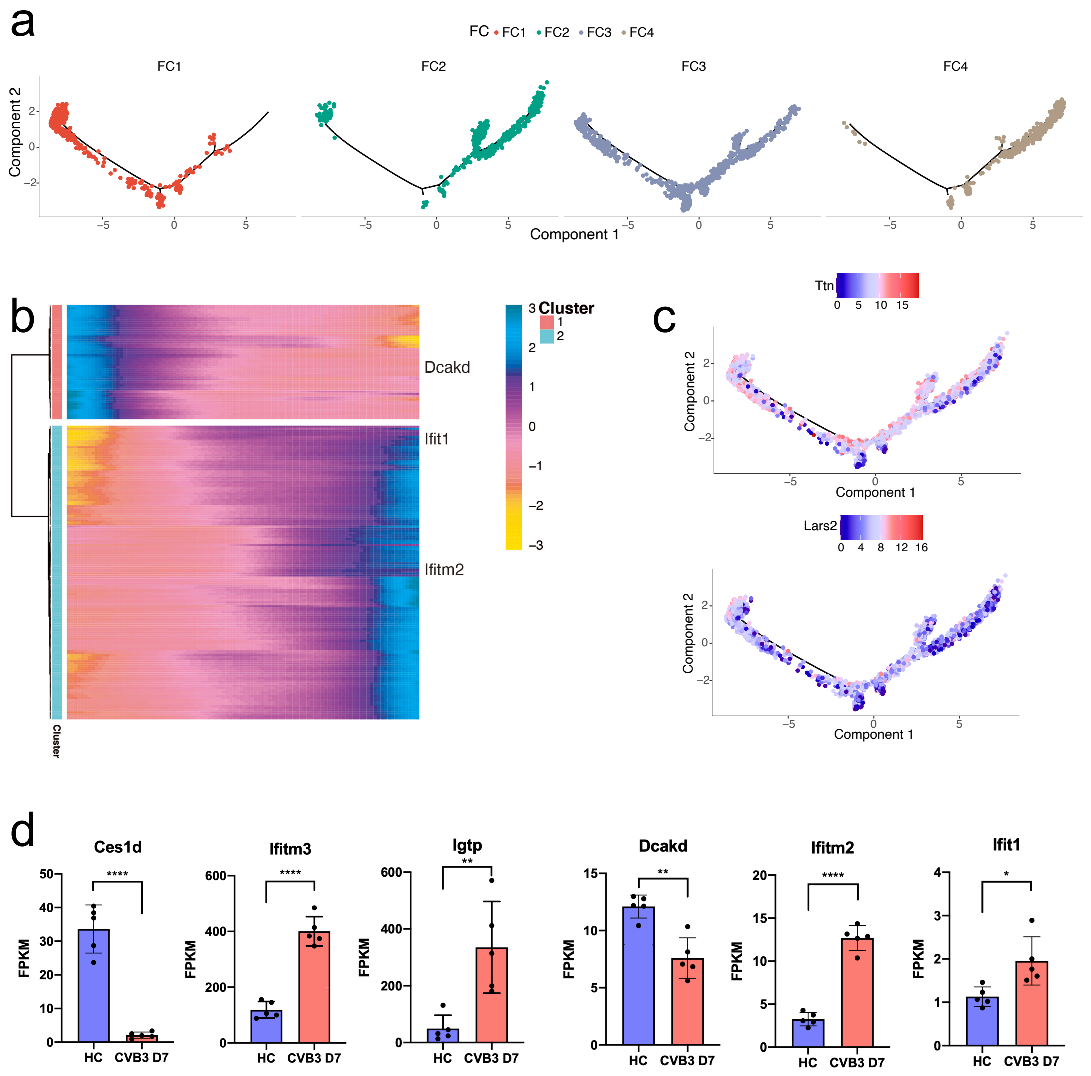

Supplementary Fig. S6 Developmental trajectory among FCs. a Developmental trajectory of FC1, FC2, FC3 and FC4, spited by FCs. **b** Expression of marker genes for each FC along the developmental trajectory. **c** Expression of marker genes for FC3 along the developmental trajectory. **d** Expression level of marker genes for each FC in bulk RNAseq data.

Supplementary Fig. S7

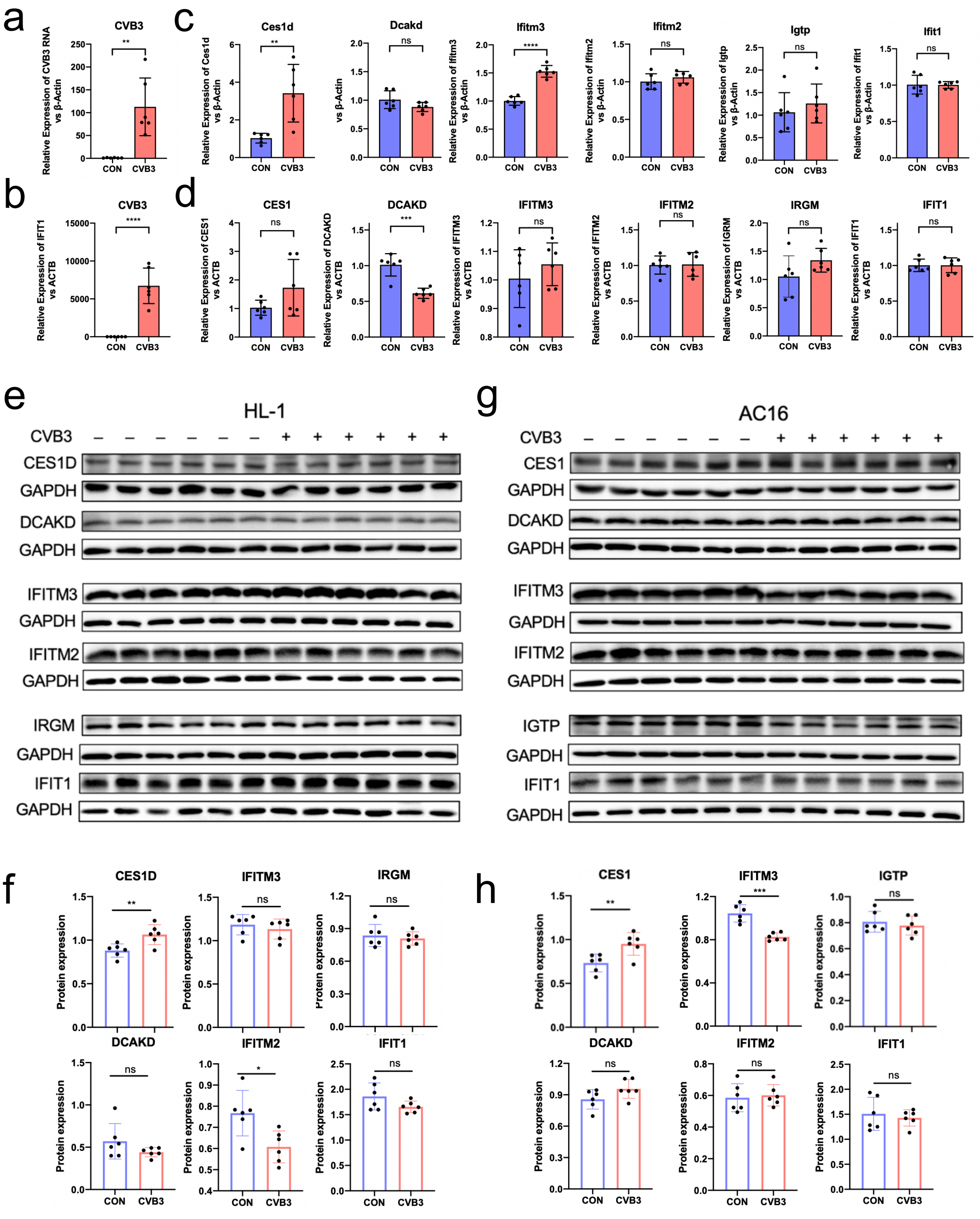

Supplementary Fig. S7 CVB3 stimulation of cardiomyocytes in vitro. The level of CVB3 in HL-1 (**a**) and AC16 (**b**) that stimulated with CVB3 in vitro. Expression level of marker genes for each FC in HL-1 (**c**) and AC16 (**d**) that stimulated with CVB3 in vitro. The protein expression level (**e**) and statistical graphs (**f**) of marker genes for each FC in HL-1 that stimulated with CVB3 in

vitro. The protein expression level (**g**) and statistical graphs (**h**) of marker genes for each FC in AC16 that stimulated with CVB3 in vitro.

Supplementary Fig. S8

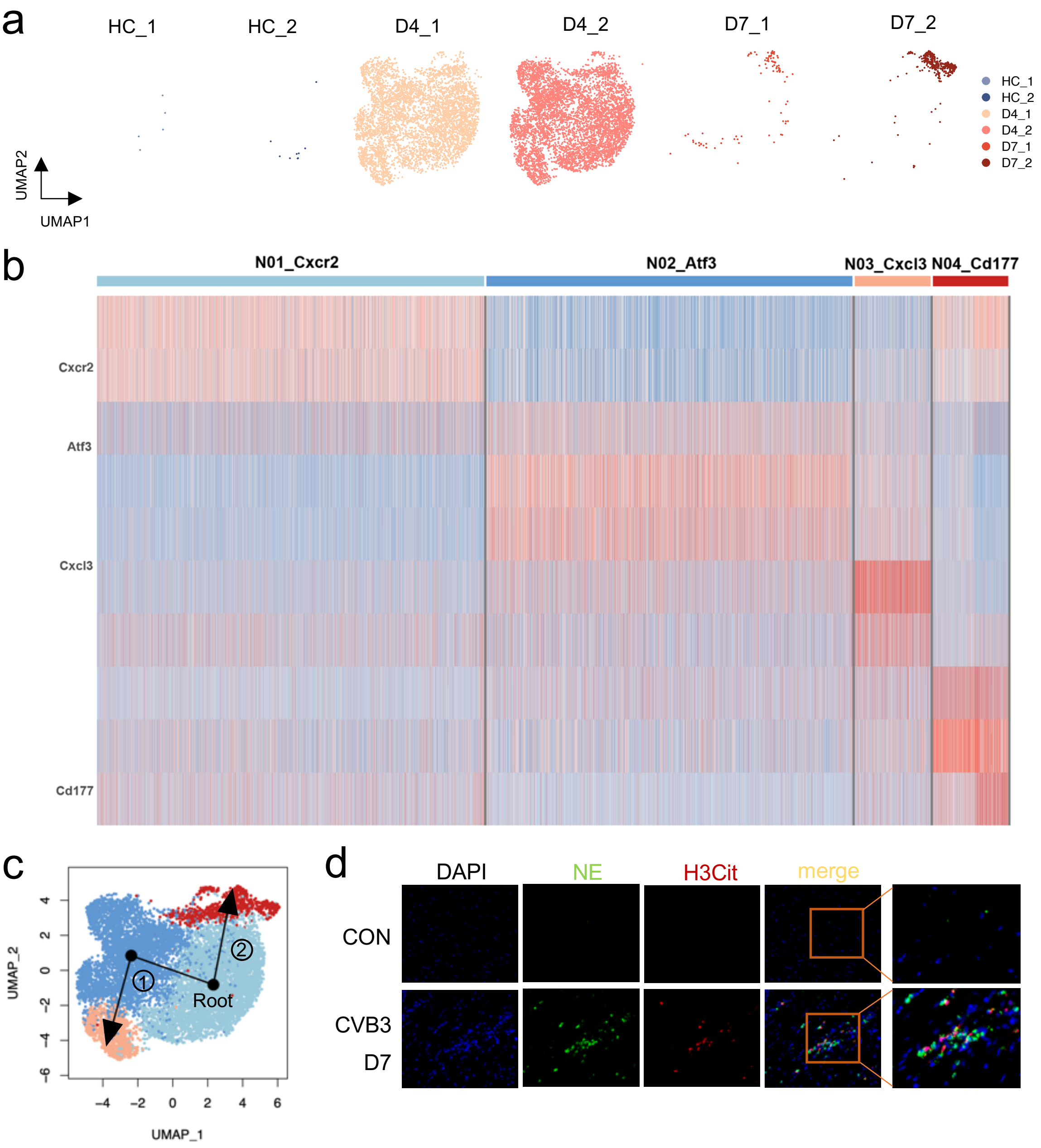

Supplementary Fig .S8 Characteristics of cardiac infiltrating neutrophils. **a** UMAP embedding of cardiac neutrophils split by samples. **b** Heatmap showing top 10 DEGs among 4 neutrophils clusters. **c** Developmental trajectory of neutrophils as revealed by SlingShot. **d** Immunofluorescence staining of neutrophil extracellular traps in the heart sample of FM mice at day 7. NE, neutrophil elastase; H3Cit, citrullinated histone H3.

Supplementary Fig. S9

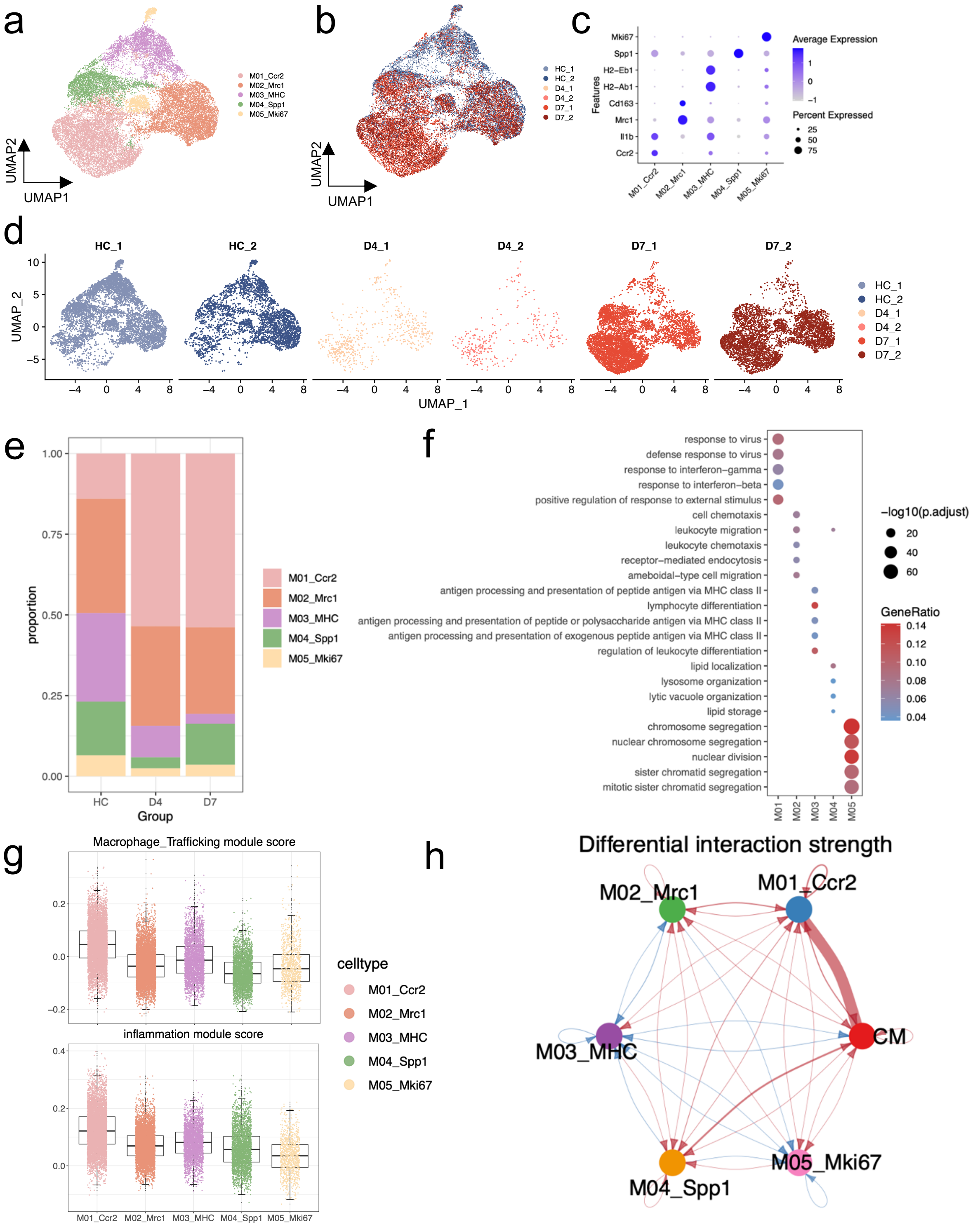

Supplementary Fig. S9 Characterization of macrophages at different stages of FM. UMAP embedding of macrophage subtypes colored by manually annotated cell types (**a**) and samples (**b**). **c** Bubble plot shows the expression levels of cell typing genes in each macrophage subtypes. Dot

color represents the gene expression level, and dot size represents the percentage of cells expressing the respective gene. **d** UMAP embedding of cardiac monocytes and macrophages split by samples. **e** Cell proportion change of cardiac macrophages and monocytes at different time points. **f** Gene ontology analysis of specifically expressed genes in each macrophage cell types. **g** Box plot of characteristic scores among different macrophage subclusters. Scores were calculated by AddModuleScore function of the Seurat package. Dots colored by cell types**. h** Strength change of ligand-receptor pairs change between any pair of two cell populations among macrophages and CMs of D7 compared to HC. The edge width was proportional to the indicated number of ligand-receptor pairs. Red represents increased in number, blue represents decreased in number.

Supplementary Fig. S10

a

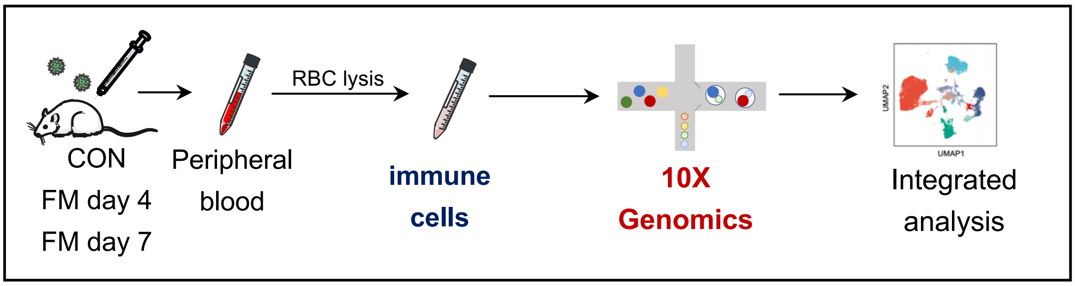

b

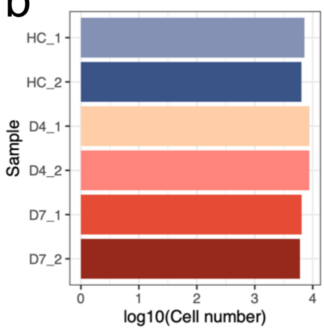

c

Sample	UMI count	Gene count
HC_1	2053	981
HC_2	2062	984
D4_1	2155	872
D4_2	2069	873
D7_1	2043	876
D7_2	1542	740

d

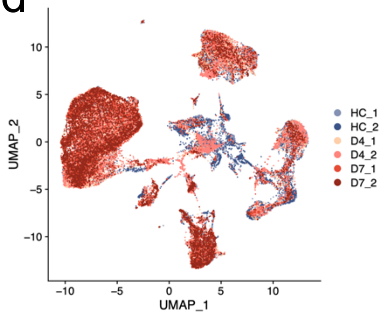

e f

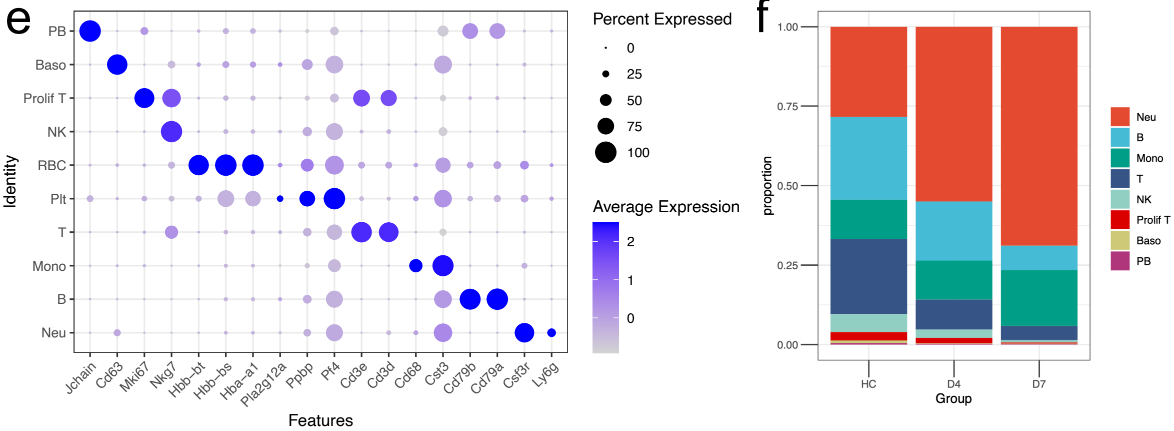

Supplementary Fig. S10 Characteristics of peripheral immune cells from FM mice at different time points. a Pipeline for prepare peripheral immune cell samples at different time points of FM. **b** Bar plot showed the $\log_{10}$ transformed cell number of each sample. **c** UMI count and gene count of peripheral immune cells in each sample. **d** UMAP embedding of peripheral immune cells from all profiled samples colored by samples. **e** Dot plot showed the expression levels of cell typing genes in each immune cell subtypes. Dot color represented the gene expression level, and dot size represented the percentage of cells expressing the respective gene. **f** Cell proportion change of peripheral immune cells at different time points.

Supplementary Fig. S11

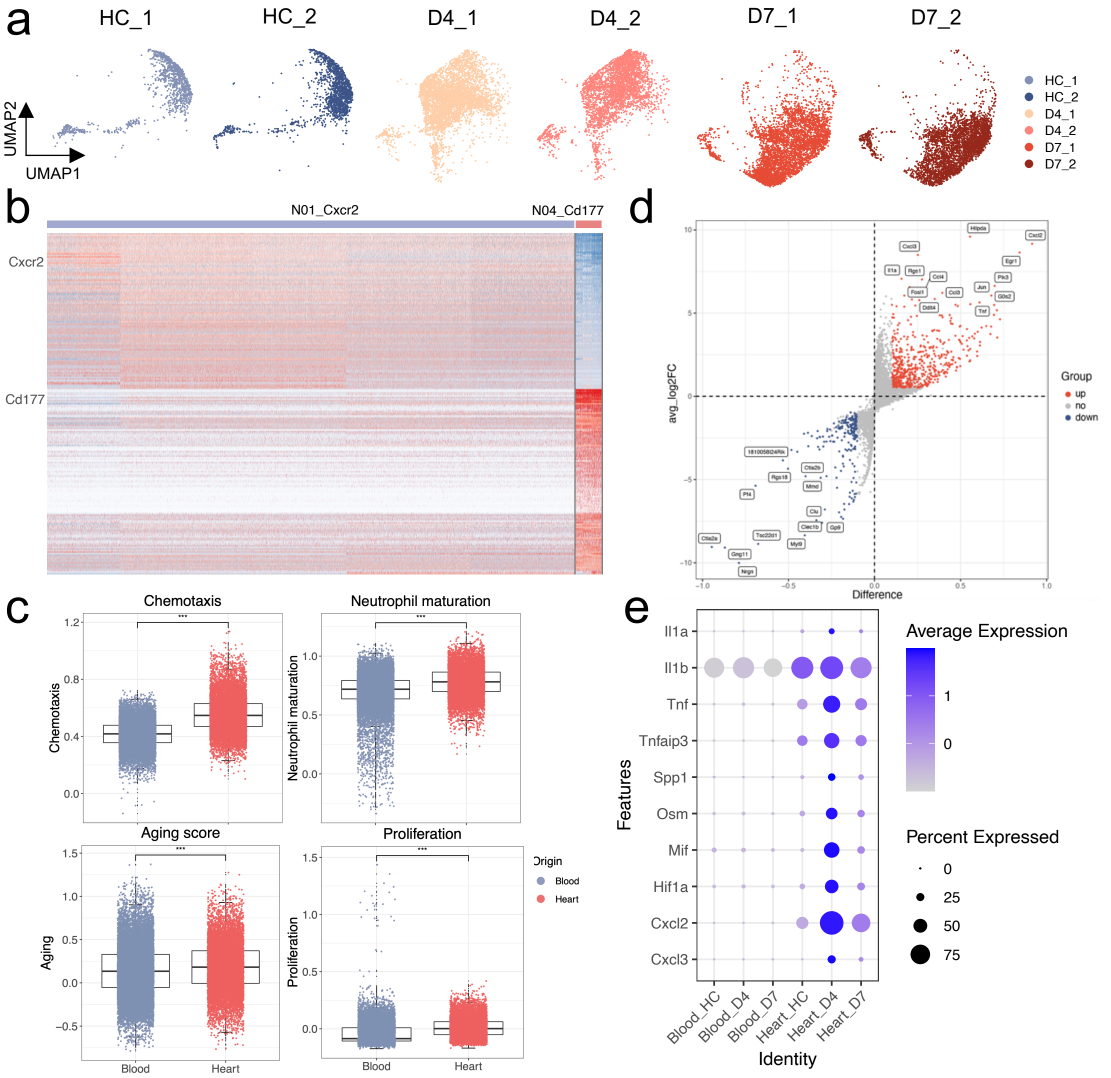

Supplementary Fig. S11 Characteristics of peripheral immune cells from FM mice at different time points. a UMAP embedding of peripheral neutrophils split by samples. **b** Heatmap showing top feature genes among peripheral neutrophil clusters. **c** Box plot of characteristic scores between cardiac and peripheral neutrophils at day 4. **d** Volcano plot of differential expression genes between cardiac and peripheral neutrophils at day 4. **e** Bubble plot shows the expression levels of cytokines in cardiac and peripheral neutrophils at different points. Dot color represents the gene expression level, and dot size represents the percentage of cells expressing the respective gene.

Supplementary Fig. S12

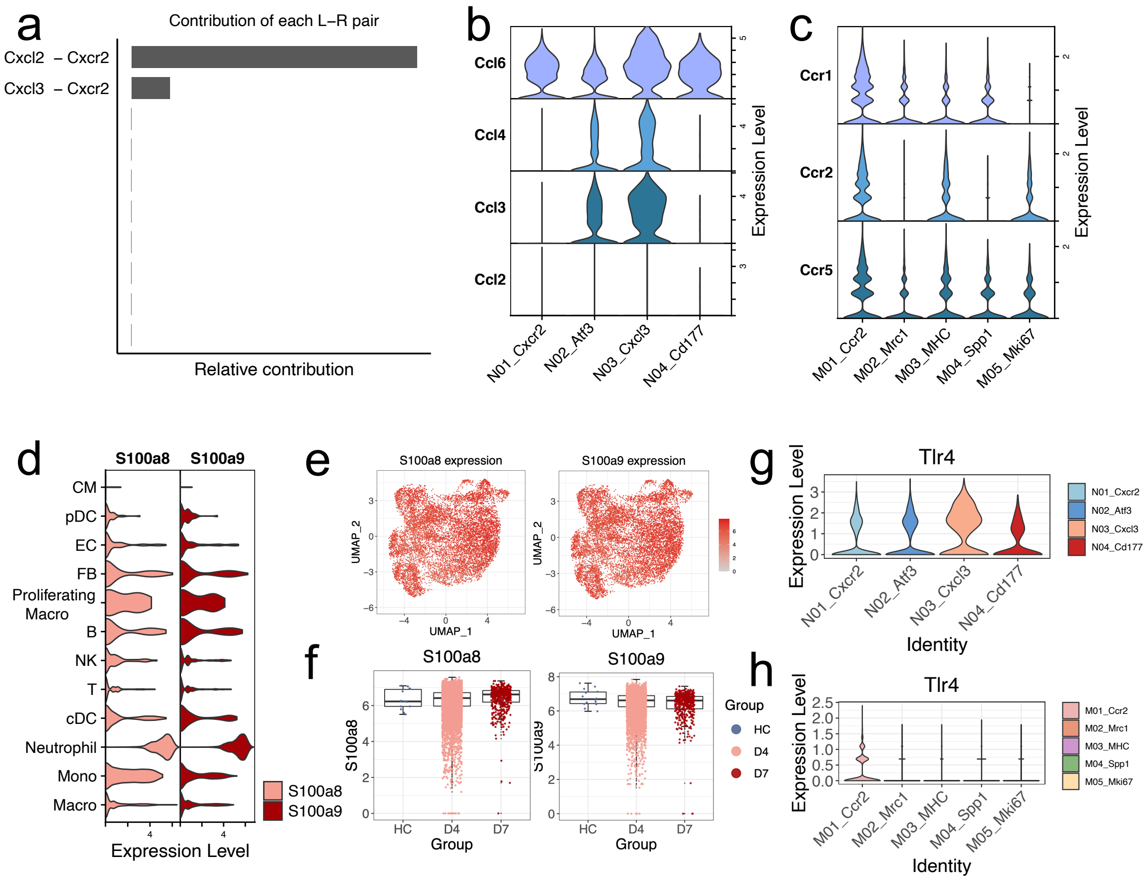

Supplementary Fig. S12 Functions of cardiac neutrophils. a Contribution of each ligand receptor pair of the CXCL family in cardiac neutrophils. **b** Violin plot shows the expression levels of chemokines of CCL family in each neutrophil subtypes. **c** Violin plot shows the expression levels of chemokines receptors of CCR family in each macrophage subtypes. **d** Violin plot shows the expression of S100a8 and S100a9 in cardiac immune cells. **e** Feature plot of S100a8 and S100a9 in cardiac infiltrating neutrophils. **f** Box plot showing the expression of S100a8 and S100a9 in cardiac infiltrating neutrophils at different time points. Violin plot showing the expression of Tlr4 in cardiac neutrophils (**g**) and cardiac macrophages (**h**).

Supplementary Fig. S13

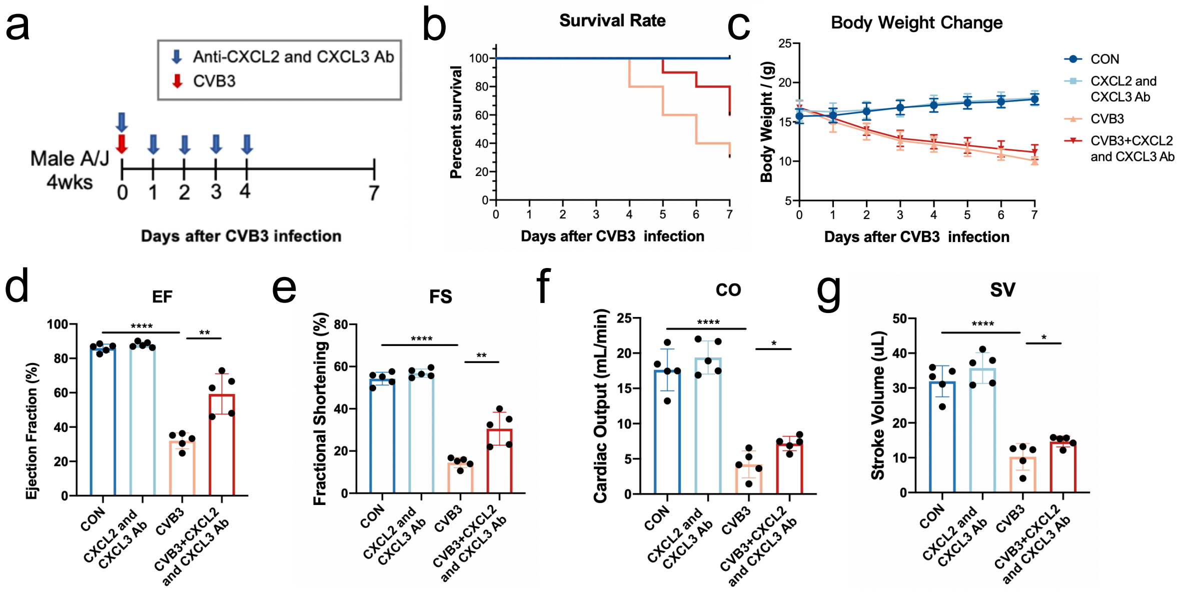

Supplementary Fig. S13 Early blockade of CXCL2 and CXCL3 decreases cardiac immune infiltration, improving cardiac function. a Experimental layout of anti-CXCL2 and CXCL3 Ab treatment. **b** Survival rate of FM mice during disease progression. **c** Body weight change of FM mice during disease progression. The ejection fraction (**d**), fraction shortening (**e**), cardiac output (**f**) and stroke volume (**g**) change of FM and CXCL2 and CXCL3 Ab treated mice.

Supplementary Fig. S14

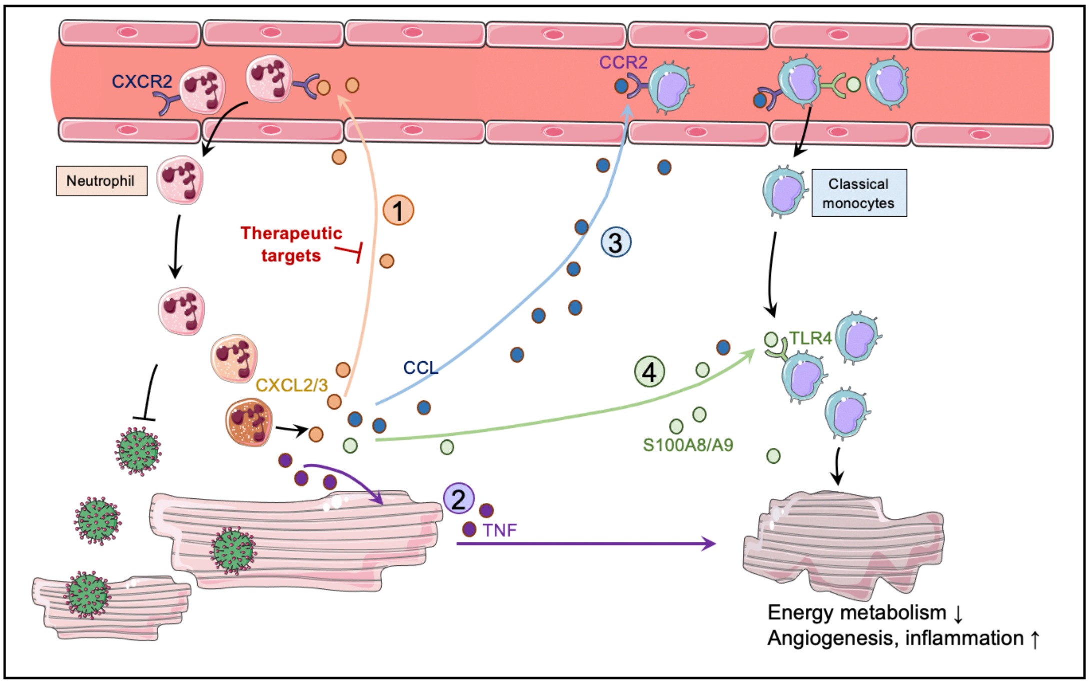

Supplementary Fig. S14 Neutrophils chemotaxis from peripheral blood at the onset of the disease and developed into pro-inflammatory phenotype. The well-developed neutrophils function as: 1. continuously attracting peripheral neutrophils through expressing high level of Cxcl2/Cxcl3; 2. promoting phenotypic change of CMs. 3. attracting peripheral monocytes through expressing chemokines of CCL family; 4. activating pro-inflammatory monocytes through releasing S100A8/A9;
